# Weakening density dependence from climate change and agricultural intensification triggers pest outbreaks: a 37-year observation of cotton bollworms

**DOI:** 10.1002/ece3.1190

**Published:** 2014-08-12

**Authors:** Fang Ouyang, Cang Hui, Saiying Ge, Xin-Yuan Men, Zi-Hua Zhao, Pei-Jian Shi, Yong-Sheng Zhang, Bai-Lian Li

**Affiliations:** 1 State Key Laboratory of Integrated Management of Pest and Rodents, Institute of Zoology, Chinese Academy of SciencesBeijing, 100101, China; 2 Department of Botany and Zoology, Centre for Invasion Biology, Stellenbosch UniversityMatieland, 7602, South Africa; 3 Fisher College of Business, Ohio State UniversityColumbus, Ohio, 43210; 4 Institute of Plant Protection, Shandong Academy of Agricultural SciencesJinan, 250100, China; 5 Department of Entomology, China Agricultural UniversityBeijing, 100193, China; 6 Institute of Bamboo, Nanjing Forestry UniversityNanjing, 210037, China; 7 College of Plant Protection, Hunan Agricultural UniversityChangsha, 410128, China; 8 Department of Botany and Plant Sciences, Ecological Complexity and Modeling Laboratory, University of CaliforniaRiverside, California, 92521-0124

**Keywords:** Climate change, density dependence, human activity, population dynamic, time series, variation partitioning

## Abstract

Understanding drivers of population fluctuation, especially for agricultural pests, is central to the provision of agro-ecosystem services. Here, we examine the role of endogenous density dependence and exogenous factors of climate and human activity in regulating the 37-year population dynamics of an important agricultural insect pest, the cotton bollworm (*Helicoverpa armigera*), in North China from 1975 to 2011. Quantitative time-series analysis provided strong evidence explaining long-term population dynamics of the cotton bollworm and its driving factors. Rising temperature and declining rainfall exacerbated the effect of agricultural intensification on continuously weakening the negative density dependence in regulating the population dynamics of cotton bollworms. Consequently, ongoing climate change and agricultural intensification unleashed the tightly regulated pest population and triggered the regional outbreak of *H. armigera* in 1992. Although the negative density dependence can effectively regulate the population change rate to fluctuate around zero at stable equilibrium levels before and after outbreak in the 1992, the population equilibrium jumped to a higher density level with apparently larger amplitudes after the outbreak. The results highlight the possibility for exogenous factors to induce pest outbreaks and alter the population regulating mechanism of negative density dependence and, thus, the stable equilibrium of the pest population, often to a higher level, posing considerable risks to the provision of agro-ecosystem services and regional food security. Efficient and timely measures of pest management in the era of Anthropocene should target the strengthening and revival of weakening density dependence caused by climate change and human activities.

## Introduction

Understanding drivers of population dynamics, including both endogenous density-dependent factors (e.g., biotic interaction) and exogenous density-independent factors (e.g., climate and human activity) (Sibly et al. 2005; Brook and Bradshaw 2006; Russell et al. 2011), is central to ecology (Berryman and Turchin 2001; Betini et al. 2013). Efficient pest management requires a fundamental understanding of how these factors interact and affect the recruitment and dynamics of the pest species (Norris et al. 2003; Kogan and Jepson 2007; Liebhold and Tobin 2008). However, such endeavors are constrained by the lack of long-term data on agricultural pest dynamics, especially given that all potential drivers (e.g., climate and agricultural practice) are constantly changing at different temporal paces. The cotton bollworm, *Helicoverpa armigera* (Hübner) (Lepidoptera: Noctuidae), characterized by its polyphagy, high mobility, high fecundity, and facultative diapause (Wu and Guo 2005), is one of the most damaging crop pests in Asia (Wu et al. 2008). It is therefore of both theoretical and practical value to examine drivers of the population dynamics for the cotton bollworm. Here, we do so by examining light-trap records from a 37-year continuous survey in eastern China that has experienced rapid changes in both climate and agricultural intensity (Wu and Guo 2005).

Density-dependent mechanisms are important drivers of population dynamics in many species (Murdoch 1994; Brook and Bradshaw 2006). Of particular importance, negative density-dependence, or density-dependent restriction, describes the decline of vital rates (growth, survival, recruitment, and reproduction) with the increase of population density, through enhanced mortality from predation and competition (Hixon and Johnson 2001). It is an essential regulator for population persistence and community stability (Churcher et al. 2006). The population dynamics of many insect species are indeed strongly regulated by density-dependent mechanisms (Stiling 1987; Ferguson and Joly 2002; Pickens 2007). For instance, density-dependent regulation explains 50% temporal variation of the *Maculinea* butterfly population (Nowicki et al. 2009). How density dependence regulates the population dynamics of the cotton bollworm is largely unknown.

Climate change is evident in the past century, from rising temperature and changing rainfall patterns to more frequent extreme weather events (Stenseth et al. 2003; Prendergast 2008). It has a profound impact on the community assemblage of insects and their interactions with host plants and natural enemies (Coley 1998; Hance et al. 2007). Indeed, climate change has been associated with the increase of pest outbreaks (Kurz et al. 2008) and the decline and/or extinction of some insect species (Thomas et al. 2004). Rising temperature and increasing annual precipitation have extended northwardly the distribution of three grasshopper species in the Inner Mongolian grassland (Guo et al. 2009). Rising minimum winter temperature also accounts for the increase in summer populations of the rice stem borer (*Chilo suppressalis*) and the green rice leafhopper (*Nephotettix cincticeps*) (Yamamura et al. 2006). However, how the interplay of climate change and density dependence affects the population dynamics of the cotton bollworm is yet unknown.

Agricultural activity has intensified across the globe, resulting in homogenized agricultural landscapes with expanding crop fields (Bianchi et al. 2006). Tillage, drainage, intercropping, rotation, grazing, and extensive usage of pesticides and fertilizers have drastically affected the flora and fauna in agricultural ecosystems (McLaughlin and Mineau 1995). These cultivation practices have been related to the population dynamics of many insect species and small burrow-dwelling mammals (Jacob 2003; Yan et al. 2012). For example, the use of the rice planting machine and combine harvester has led to the decline of the rice stem borer via killing its larvae when cutting rice stems (Yamamura et al. 2006). Habitat deterioration and loss due to intensified agricultural activities are also responsible for the decline of butterfly populations in Europe (van Swaay et al. 2006; Van Dyck et al. 2009). Agricultural irrigation with increasing per capita production of crops has also changed the population growth of rodents in East Asia (Yan et al. 2012). Nevertheless, studies on how agricultural activities interact with density dependence and climate change in regulating the population dynamics of insect pests are rare.

The use of long-term data is essential for elucidating the regulating mechanism of population dynamics (Bjornstad and Grenfell 2001). One such data type available to pest managers is time series, recording the relative abundance of a focal species collected at a regular interval (Berryman and Turchin 2001). Time-series analysis can address whether there are systematic population trends or discontinuities due to exogenous forces (Bjornstad and Grenfell 2001). It can also be used to determine which dynamic behaviors the population follows (e.g., asymptotically stable / periodic or chaotic fluctuation) and how accurately we can forecast population dynamics. Answers to these questions provide insights to assessing the relative importance of the endogenous factor (i.e., density dependence) and exogenous density-independent (often top-down) variables in regulating population dynamics (Berryman 1991; Hastings et al. 1993). To ensure the provision of agro-ecosystem services, it is necessary to elucidate the regulation mechanism of *H. armigera* using the time-series analysis and to quantify how the interplay of density dependence, climate change, and agricultural activity regulates the population dynamics of cotton bollworms.

Based on long-term time-series data collected since 1975, we here examine how the population change rate of the cotton bollworm is determined by both the endogenous factor of density dependence and the exogenous factors of climate and agricultural activity. To do so, we sequentially address four interrelated issues:

1. detect long-term trends of the regional cotton bollworm population;
2. identify the strength and form of density dependence at both the annual and generational scales (four generations per annum);
3. determine main drivers of the population change rate of *H. armigera* and the potential interactions between endogenous and exogenous drivers; and
4. quantify the relative contribution of these identified drivers in explaining the variation of population change rate. Such knowledge is essential for the success and efficiency of regional pest management which then ensures the service provision in agro-ecosystems.

## Materials and Methods

### Data capture

**Figure 1:**
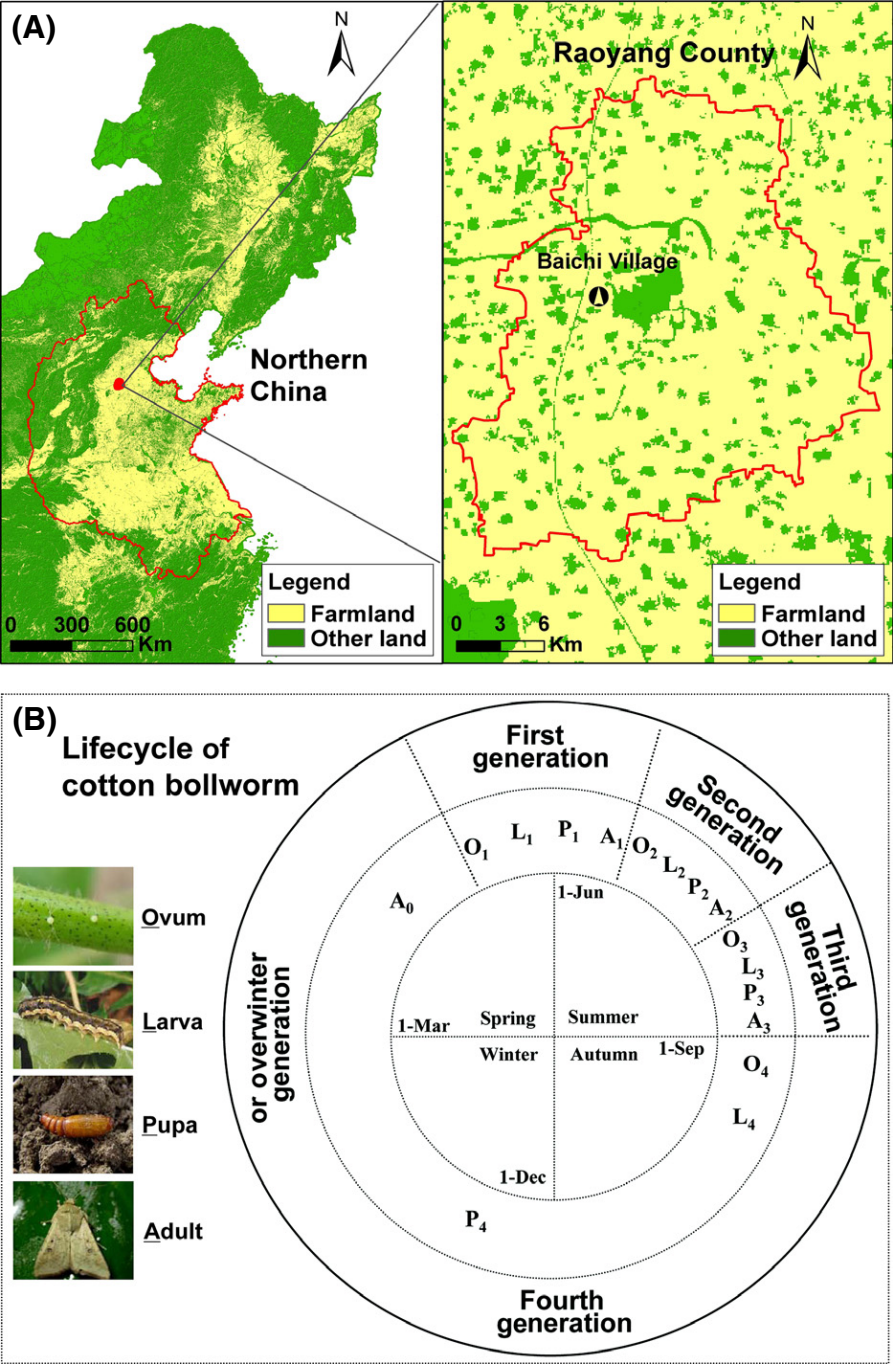
The study region and the lifecycle of the cotton bollworm. (A) Maps of land use in northern China (left) and in Raoyang County (right). The monitoring site was set in Baichi Village. (B) The four generations of the cotton bollworm and four distinct seasons in the region.

The study area is located at Raoyang County (38°05′–38°20′N, 115°34′–115°55′E) in Hebei Province of China (Fig.1A), with a warm temperate climate of four distinct seasons (Fig.1B) and farmlands being the dominant land use. Major crops in the study area include wheat, corn, peanut, cotton, soybean, sorghum, and cole. Local daily temperature (°C) and precipitation (mm) were obtained from the China Meteorological Data Sharing Service System (http://cdc.cma.gov.cn). Total crop yield (ton), irrigation area (ha), plowed area (ha), and the quantity and mechanomotive force of agricultural machinery during 1980–2010 were obtained from the Agricultural Information Institute of the Chinese Academy of Agriculture Sciences. Irrigation area was highly correlated with plowed area (*r* = 0.825, *P* < 0.001), the quantity (*r* = 0.872, *P* < 0.001) and mechanomotive force (*r* = 0.741, *P* < 0.001) of agricultural machinery, and with total crop yield (*r* = 0.776, *P* < 0.001). Consequently, we only used the irrigation area to represent agricultural activity in the following analyses to avoid the multicollinearity.

The cotton bollworm has four generations per year in the region (Fig.1B) (Ge et al. 2005). The first-generation larvae mainly target wheat as the host crop, while other three generations target all major crops (Wu and Guo 2005). Adult abundance of *H. armigera* has been surveyed using light traps at night since 1975. Trapping sites at Raoyang County were located at Wugong Village before 2001 and moved to Baichi Village afterward, 10 km apart (Fig.1A). Trapping started at dusk (1800 h local time) and finished at dawn (0600 h) every day from the beginning of April to the end of September each year from 1975 to 2011. The adults of the four generations were counted daily. We used the annual total abundance and the abundances of the four generations in the following analysis.

### Time-series analysis

We calculated the realized population change rate of *H. armigera* (Turchin 1999) using the R-function (Berryman and Turchin 2001), *R*_*t*_ = Ln(*N*_*t*_/*N*_*t*−1_), where *N*_*t*_ and *N*_*t*−1_ are the total abundance of captured adults in year *t* and *t*−1, respectively. Using the partial autocorrelation function (PACF) (Sequeira and Dixon 1997; Berryman and Turchin 2001), we found that the annual population dynamics of the cotton bollworm showed a clear first-order feedback, that is, the current population size is only related to the population size in the previous year (Fig. S1a), with 16% variation explained. The influence of higher-order feedbacks (i.e., the population sizes of two or 3 years ago) on the current population size is trivial, with less than 3% accumulated variation explained (Fig. S2a). Consequently, we only considered the time lag of 1 year in the annual autoregressive model. Specifically, the population change rate (*R*) was used as the dependent variable, with logarithmically transformed abundance (*X* = Ln(*N*)) at year *t*−1, temperature (*T*), precipitation (*P*), and irrigation area (*I*) at year *t* as explanatory variables. To detect the effect of nonlinearity, we selected the generalized additive model (GAM) for the autoregression (Hastie and Tibshirani 1990), using the natural cubic splines with a maximum of four knots (Wood 2006b). The GAM for the annual population dynamics reads:


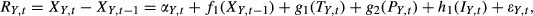
(1)

where the subscript *Y* indicates that the model is for the annual total population size, differentiating from the seasonal models below for specific generations of *H. armigera* within the year; *T*_*Y,t*_ the annual average of daily temperature in year *t*; *P*_*Y,t*_ the annual total precipitation in year *t*; *I*_*Y,t*_ the annual irrigation area in year *t*; and *α*_*Y,t*_ and *ε*_*Y,t*_ are intercept and normally distributed stochasticity. Meanings of all dependent variables, explanatory variables, and parameters of model (1) are provided in Table S1.

We also developed autoregressive models for the four generations of the cotton bollworm (Fig.1B). Logarithmic adult abundances of the overwintering, first, second, and third generations for year *t* are designated as *X*_*O,t*_, *X*_*F,t*_, *X*_*S,t*_*,* and *X*_*T,t*_, respectively (Fig.1B). According to the PACF, the population dynamics of within-year generations also showed predominantly a first-order feedback (Fig. S1b), with 43% variation explained by the abundance of previous generation (Fig. S2b). Other higher-order feedbacks only explained less than 3% variation (Fig. S3b) and were thus ignored in the autoregressive models for the four generations. As the overwintering generation needs to endure a harsh winter, we examined four specific temperature variables separately in the autoregressive model for this generation; specifically, we considered the temperature (*T*_*O,t*_) in model (2) to represent the mean, minimum, maximum, and the below-zero total of daily temperature, respectively, during the overwintering period. For the four generations of the cotton bollworm, we have the following autoregressive GAM models:


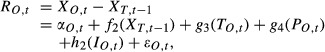
(2)

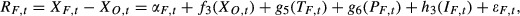
(3)

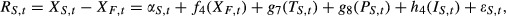
(4)

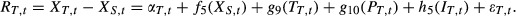
(5)

Meanings of all dependent variables, explanatory variables, and parameters of models (2–5) are also provided in Table S1.

Analyses were carried out in R version 2.15.2 using the MGCV package version 1.7–22 (Wood 2006a). The optimal roughness of the smoothing term was determined by minimizing the generalized cross-validation value (GCV) (Stige et al. 2006). The best-fit model was also selected from 2^*n*^−1 candidate models (where *n* is the number of explanatory variables) by minimizing the GCV provided that all variables were statistically significant (*P* < 0.05) for inference (Cox et al. 1981) (Tables S2 and S3). Residuals from the annual and generational GAMs followed approximately normal distributions and showed no significant autocorrelation (Fig. S3). As an alternative method, we also analyzed the population dynamics using general linear models (LMs), with the functions *f*_*i*,_ *h*_*i*_, and *g*_*i*_ in the GAMs replaced by linear relations and pairwise interactions of explanatory variables (Table S2 and S4).

To test the possible interactions among density dependence, climate, and human activity, we used the tensor product smoothing (TPS) method (Wood 2006a) and the moving-window (MW) method (Fig. S5) (Yan et al. 2012). First, different combinations of exploratory variables in the above models were smoothed by the TPS method at the annual and generational timescales using the MGCV package in R (Wood 2006b), with significant terms (at *P* < 0.05) further used for inference. Second, we selected a time window (from 7 to 12 years) and calculated the mean for each explanatory variable within the window. We estimated the density dependence coefficient *b* from the linear regression, *R* = *a* + *b* • *X*, and then tested the significance of correlation coefficients between *b* and the average temperature, precipitation or irrigation area for the given time window using a linear regression model. The interactions between other significant variables were tested the same way using linear regression models (Yan et al. 2012). For instance, to calculate interaction between temperature and precipitation (both significant variables) for the third generation, we first estimated the effect of temperature on the population change rate (*b*) from the linear regression, *R*_*T,t*_ = *a* + *b* × Temperature_*T,t*_, and then tested the significance of correlation coefficients between *b* and the precipitation for a given time window using linear regression models.

To clarify the role of each explanatory variable in regulating the population dynamics in the cotton bollworm, we partitioned the variation of its annual and generational population change rates (*R*) using variation partitioning (VP) (Borcard et al. 1992). Specifically, the variation of the population change rate was partitioned according to three sets of variables into eight independent components: density dependence (Denci), climate (Clima, including temperature and precipitation), human activity (Irrig: irrigation area), interactions of density dependence and climate (Denci × Clima), climate and human activity (Clima × Irrig), dependence and human activity (Denci × Irrig), the intersection of all three sets (Denci × Clima × Irrig), and the unexplained variation (Residuals). VP was implemented in VEGAN package in R (Oksanen 2008).

## Results

### Annual population dynamics

**Figure 2:**
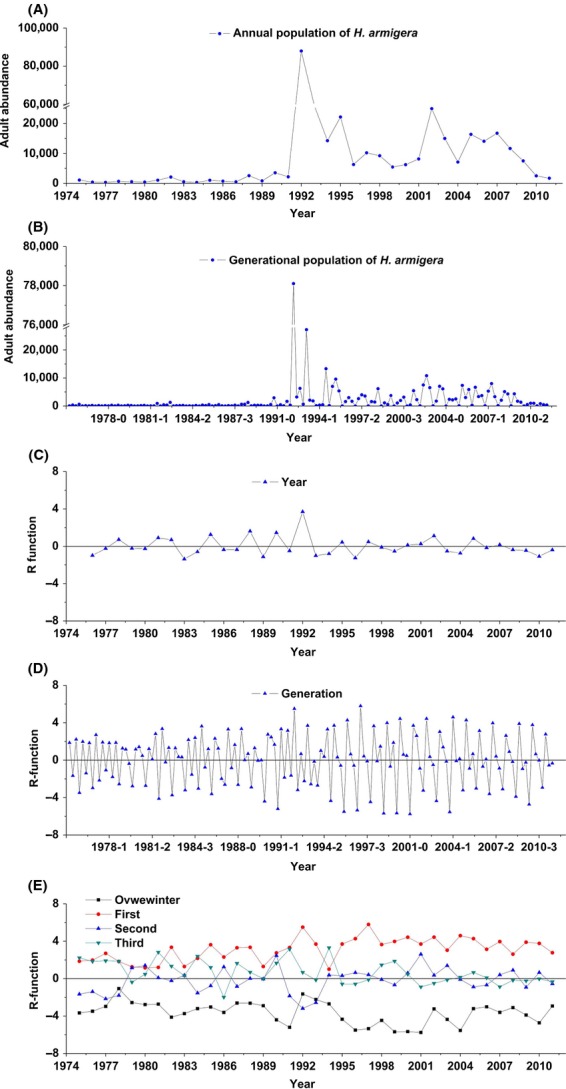
The dynamics of population abundance and population change rate (R-function) for the cotton bollworm. (A) The annual and (B) generational dynamics of population abundance of cotton bollworms from 1975 to 2011. (C) The annual and (D) generational population change rate (R-function) of cotton bollworms. (E) The population change rate (R-function) for the overwintering, first, second, and third generations.

The annual abundance of the cotton bollworm reached its peak in 1992 (Fig.2A). The mean annual abundance of captured adults after 1992 was 11 times more than that before 1992 (mean ± SD: 12173.3 ± 7811.9 vs. 1099.2 ± 925.2; *t*_34_ = 5.8, *P* < 0.001). In contrast, the population change rate (*R*_*Y,t*_ = 0.012 ± 1.008) fluctuated around zero in the annual population dynamics from 1975 to 2011 (*t*_35_ = 0.074, *P* = 0.942; Fig.2C), also for periods before and after 1992 (0.043 ± 0.942, *t*_16_ = 0.183, *P* = 0.857 for before 1992; −0.207 ± 0.656, *t*_36_ = −1.379, *P* = 0.185 for after 1992; Fig.2C). No significant difference was found between the annual population change rates (*R*_*Y,t*_) before and after 1992 (*t*_1,33_ = 0.924, *P* = 0.362).

**Figure 3:**
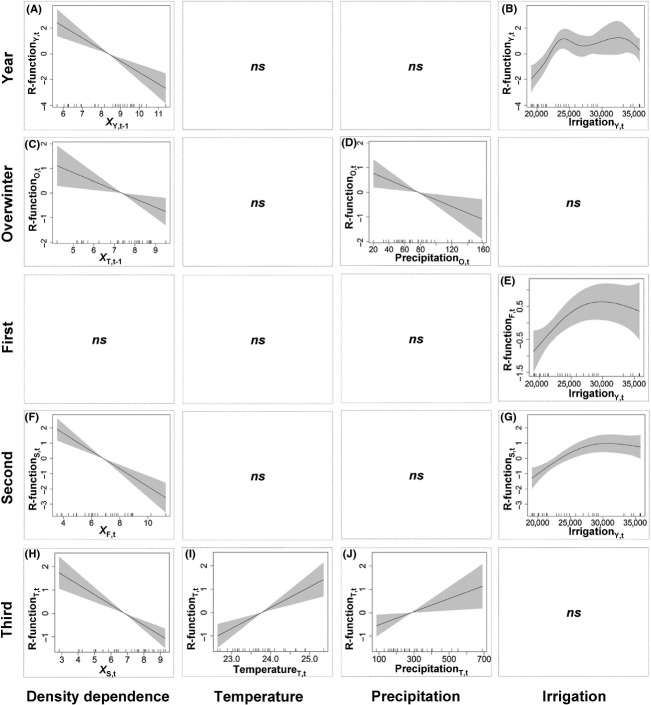
Main effects of endogenous and exogenous factors on the population change rate of annual and four generations of adult cotton bollworms from the generalized additive models. Shade indicates the 95% confidence band; ns, non significant effect. Partial effects of density in last a year (A) and irrigation area (B) on the annual population change (R-function_*Y,t*_); partial effects of the third generation density in last year (C) and the precipitation during period of the overwinter generation (D) on the overwinter generation population change (R-function_*O,t*_); partial effects of irrigation area (E) on the first generation population change (R-function_*F,t*_); partial effects of the first generation density (F) and irrigation area (G) on the second generation population change (R-function_*S,t*_). partial effects of the second generation density (H), the temperature during period of the third generation(I), and the precipitation during period of the third generation (J) on the third generation population change (R-function_*T,t*_).

**Figure 4:**
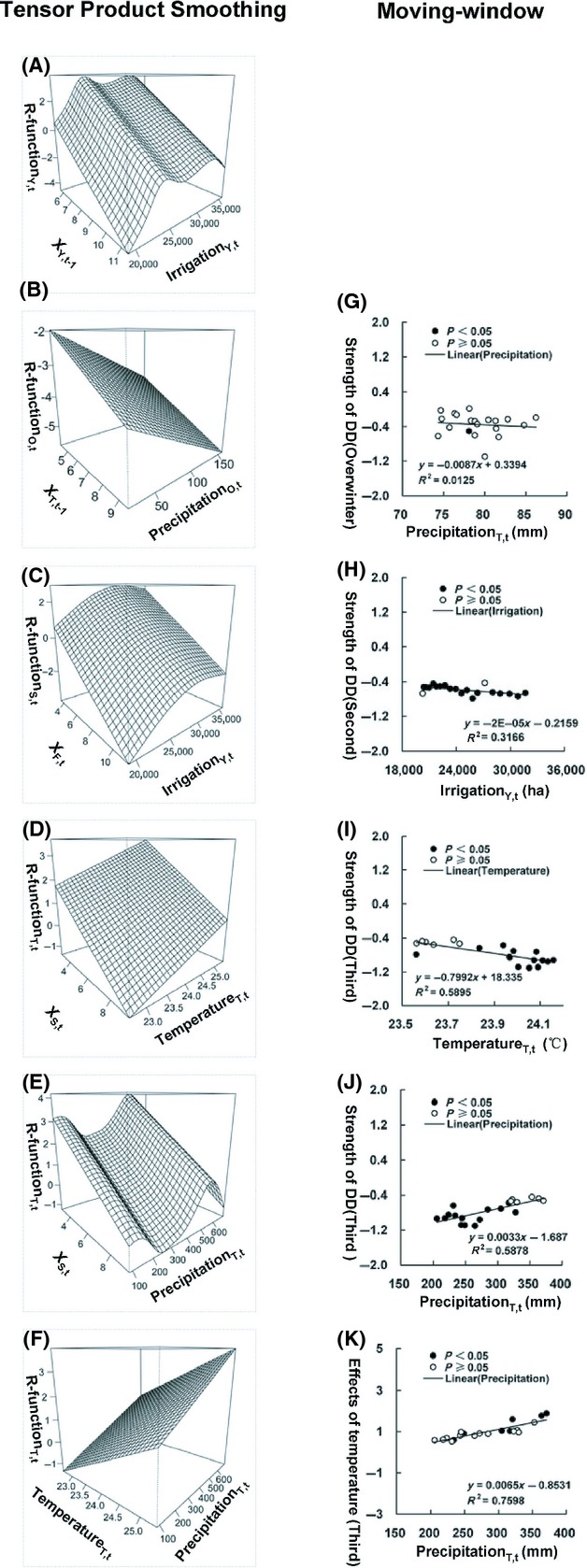
Significant interactions between endogenous and exogenous factors on population change rate of cotton bollworms from the tensor product smoothing method (left panel) and the moving-window method (right panel). The linear regression on the right panel is based on a 12-year moving-window analysis (A–F, see Methods; solid dots, *P* < 0.05; circles, *P* ≥ 0.05). The interactive effects of density in the whole last year (*X*_*Y*′*,t*−1_) and irrigation area (Irrigation_*Y,t*_) on the annual population change (R-function_*Y,t*_) (A); the interactive effects of the third generation density in last year (*X*_*T*,*t*−1_) and the precipitation during period of the overwinter generation (Precipitation_*O,t*_) on the overwinter generation population change (R-function_*O,t*_) (B); the interactive effects of the first generation density (*X*_*F,t*_) and irrigation area (Irrigation_*Y,t*_) on the second generation population change (R-function_*S,t*_) (C); the interactive effects of the second generation density (*X*_*S,t*_) and the temperature during period of the third generation (Temperature_*T,t*_) on the third generation population change (R-function_*T,t*_) (D); the interactive effects of the second generation density (*X*_*S,t*_) and the precipitation during period of the third generation (Precipitation_*T,t*_) on the third generation population change (R-function_*T,t*_) (E); the interactive effects of the temperature (Temperature_*T,t*_) and the precipitation during period of the third generation (Precipitation_*T,t*_) on the third generation population change (R-function_*T,t*_) (F), predicted from tensor product smooths respectively. Interactions among density dependence (DD), climate and irrigation. In the best-fit LM of the annual population only one explanatory variable (density) was a statistically significant term (at *P* < 0.05), then the result from the moving windows was not given (G–K); strength of DD for the overwinter generation and the precipitation during period of the overwinter generation (Precipitation_*O,t*_) (G); strength of DD for the second generation and irrigation area (Irrigation_*Y,t*_) (H); strength of DD for the third generation and the temperature during period of the third generation (Temperature_*T,t*_) (I); strength of DD for the third generation and the precipitation during period of the third generation (Precipitation_*T,t*_) (J); the effects of the temperature (Temperature_*T,t*_) on the third generation R-function and the precipitation (Precipitation_*T,t*_) (K).

Temperature and irrigation area showed significant increases during the survey period (Fig. S4a,c), while the precipitation showed a slight decline (Fig. S4b). The best-fit GAM revealed a linear negative density dependence (*F*_1,4.63_ = 15.73; *P* < 0.001; Fig.3A) and a nonlinear positive effect of irrigation area (*F*_2.63,4.63_ = 3.35; *P* = 0.035; Fig.3B, Table1) on the annual population change rate, with 41% variation explained by these two variables (Table S2). The main effects of the LM also showed a negative effect of density (*X*_*Y,t*−1_) on the annual population change rate, *R*_*Y,t*_ = 2.39–0.28*X*_*Y*_*,*_*t*−1_ (*F*_1,29_ = 5.39; *P* = 0.028), with 16% variance explained (Table S2). However, the LMs did not detect the positive effect of irrigation area on the annual population change rate. The TPS method showed that the strength of density dependence (*b*) declined nonlinearly with the increase of irrigation area (Fig.4A). In the best-fit LM, the previous year abundance was the only statistically significant term (*P* < 0.05), and thus, no interactions were reported.

**Table 1:** The effects of density dependence, temperature, precipitation, and irrigation on annual and generational population change rate of the cotton bollworm, *Helicoverpa armigera*, according to the generalized additive models.

Population change rate	Density dependence	Temperature	Precipitation	Irrigation
Annual population	–	ns	ns	+
Overwinter generation	−	ns	−	ns
First generation	ns	ns	ns	+
Second generation	–	ns	ns	++
Third generation	–	+++	+	ns

+/− indicates positive/negative effects; number of signs (from one to three) indicates the significance level at *P* < 0.05, *P* < 0.01 and *P* < 0.001; ns: not significant.

### Dynamics of four generations

The generational abundance of the cotton bollworm reached its peak in the second generation of 1992 (Fig.2B). The mean generational abundance after the second generation of 1992 was 11 times more than before (3087.4 ± 3999.3 vs. 275.4 ± 452.1; *t*_1,145_ = −5.806, *P* < 0.001). In contrast, the generational population change rate (*R*_*Y,t*_) fluctuated around zero from 1975 to 2011 (−0.012 ± 2.728, *t*_146_ = −0.056, *P* = 0.956), also for generations before and after the second generation of 1992 (−0.105 ± 2.402, *t*_68_ = −0.364, *P* = 0.717; 0.029 ± 2.985, *t*_76_ = 0.085, *P* = 0.932; Fig.2D), with no significant difference (*t*_144_ = −0.297, *P* = 0.767).

For the overwintering generation, its population change rate (*R*_*O,t*_) fluctuated below zero (−3.665 ± 1.183, *t*_36_ = −18.845, *P* < 0.001; Fig.2E). The best-fit GAM for the overwintering generation showed a linear negative density dependence (*F*_1,3_ = 7.37; *P* = 0.011; Fig.3C) and a linear negative effect of precipitation (*F*_1,3_ = 7.32; *P* = 0.011; Fig.3D), with 36% variation explained (Table S2). Consistently, the main effects of the LMs also showed negative effects of both density (*X*_*T,t*−1_) and precipitation (Prec_*O,t*_) on the population change rate of the overwintering generation, *R*_*O,t*_ = −0.204–0.353*X*_*T,t*−1_–0.013Prec_*O,t*_ (*F*_2,28_ = 7.86; *P* = 0.002), with 36% variance explained (Table S2). A significant interaction between the total below-zero temperature and precipitation was also detected by the LM (*F*_2,28_ = 8.29; *P* = 0.002), showing a slightly higher variance explained (37%) when replacing precipitation with the interaction in the above LM. The TPS method also showed an increase in the strength of density dependence with precipitation (Fig.4B).

For the first generation, its population change rate (*R*_*F,t*_) fluctuated above zero (3.108 ± 1.227, *t*_36_ = 15.411, *P* < 0.001; Fig.2E). The best-fit GAM showed a nonlinear positive effect of irrigation area (*F*_1.87, 2.87_ = 3.88; *P* = 0.029; Fig.3E), with 26% variation explained (Table S2). The main-effect LM showed a similar result of the positive effect of irrigation area (*F*_2,28_ = 4.23; *P* = 0.049), with 13% variation explained (Table S2). In contrast, three significant interactions (density × temperature, temperature × irrigation area, precipitation × irrigation area) were detected by the LM (*F*_5,25_ = 2.93; *P* = 0.04), together with density explaining 31% of the variation (Table S5). In the best-fit GAM and LM, only one explanatory variable (irrigation area) was statistically significant (*P* < 0.05); consequently, no results from the MW and TPS methods were shown.

For the second generation, its population change rate (*R*_*O,t*_) fluctuated near zero (−0.175 ± 1.298, *t*_36_ = −0.821, *P* = 0.417; Fig.2E). The best-fit GAM revealed a linear negative density dependence (*F*_1,4.03_ = 28.34; *P* < 0.001; Fig.3F) and a nonlinear positive effect of irrigation area (*F*_2.03,4.03_ = 7.08; *P* = 0.002; Fig.3G), with 54% variation explained (Table S5). The best-fit main-effect LM showed a negative effect of density dependence and a positive effect of irrigation area, with 40% variation explained (Table S2). LMs also identified a significant negative interaction between the density and irrigation area (*F*_3, 27_ = 10.57; *P* < 0.001; Table S5). The MW analysis showed a decrease of the strength of density dependence with the increase of irrigation area (Fig.4H; also see Fig. S7), consistent with the result from the TPS method (Fig.4C).

For the third generation, its population change rate (*R*_*T,t*_) fluctuated near zero (0.682 ± 1.245, *t*_36_ = 3.336, *P* = 0.002; Fig.2E). The best-fit GAM revealed a linear negative density dependence (*F*_1,4_ = 25.85; *P* < 0.001; Fig.3H), a linear positive effect of temperature (*F*_1,4_ = 15.21; *P* < 0.001; Fig.3I), and a linear positive effect of precipitation (*F*_1,4_ = 5.603; *P* = 0.025; Fig.3J), with 65% variation explained (Table S2). The best-fit main-effect LM showed a similar result (Table S2). A negative interaction between density and temperature, and a positive interaction between density and precipitation were also identified by the LM (*F*_4,26_ = 17.29; *P* < 0.001; Table S5). The MW analysis showed that the strength of density dependence declined with temperature (Fig.4I; also see Fig. S8) but increased with precipitation (Fig.4J; also see Fig. S9), consistent with the results from the TPS method (Fig.4D and E). Moreover, the effect of temperature on the population change rate was increased with the increase of precipitation (Fig.4K; also see Fig. S10, Supporting information), similar to the result of the TPS analysis (Fig.4F).

### Variance partitioning

The variance partitioning (VP) of the annual population change rate (*R*_*Y,t*_) showed the dominant effect of density dependence, explaining 20% of the total variation (Table2); climate and human activity played a trivial role in the population regulation. For the overwintering generation, density dependence and climate each explained, independently, 17% of the total variation. For the first generation, the total variation was mainly explained by climate (7.5%) and human activity (19.9%). For the second generation, density dependence accounts for 37% of the total variation, followed by human activity (18%). For the third generation, density dependence accounts for 25% of the total variation, followed climate (20%) (Table2).

**Table 2:** Variation partitioning of the population change rate according to three sets of variables into eight independent components: density dependence (Denci), climate (Clima, including temperature and precipitation), human activity (Irrig: irrigation area), interactions of variables.

	Explained variation				
	
Variables	All the year (%)	Overwinter (%)	First (%)	Second (%)	Third (%)
Denci	20.12	16.80	0.00	37.00	25.30
Clima	0.06	17.16	7.46	0.00	19.55
Irrig	0.00	0.00	19.93	17.75	0.00
Denci × Clima	0.00	0.00	0.00	0.61	4.10
Clima × Irrig	0.07	0.00	0.00	0.00	0.00
Denci × Irrig	0.00	1.11	0.00	0.00	9.25
Denci × Clima × Irrig	0.93	2.25	2.10	0.99	3.28
Residuals	78.82	62.67	72.60	44.65	41.80

## Discussion

### Negative density dependence

The cotton yield in northern China declined significantly in the early 1990s due to the outbreak of cotton bollworms (Wu and Guo 2005), consistent with our results showing that the annual abundance of the cotton bollworm reached its peak in 1992 (Fig.2A). The population change rate fluctuated around zero both before and after 1992, suggesting that the population shifted from a lower equilibrium to a much higher one in 1992. Although the large-scale cultivation of transgenic cotton from 1997 to 2006 in the region has caused damage to the ovular and larval populations of *H. armigera* in the second and third generations (Wu et al. 2008), the generational abundance of adult *H. armigera* still fluctuated at a much higher level after 1992 (Fig.2B), suggesting that major crops in northern China still face considerable risks from the dense population of *H. armigera*.

Density dependence occurs when the population change rate responds to previous population sizes (Hixon and Johnson 2001). Survival and fecundity usually decline with the increase of population density (Lorenzen and Enberg 2002; Nicoll et al. 2003) but could also decrease when the population density is low, which is known as the Allee effect (Liebhold and Tobin 2008; Tobin et al. 2011). Negative density dependence in particular is an important stabilizer (Weisberg and Reisman 2008) and regulator of animal population dynamics (Turchin 1999; Lande et al. 2002; Sibly et al. 2005; Brook and Bradshaw 2006; Betini et al. 2013). Previous studies have exemplified the relationship between negative density dependence and population change rate using time series of insects, birds, fish, and mammals (Sibly et al. 2005). Our results provide further quantitative evidence on how negative density dependence regulates the population dynamics of the cotton bollworm. Negative density dependence explained 17% variation in the overwintering generation, null in the first generation, but jumped to 37% and 25% variation explained in the second and third generations. Evidently, stronger negative density dependence suppressed the population change rate to close to zero in the second and third generations, while the population change rate deviated from zero when the negative density dependence was weak in the overwintering and first generations (Fig.2E). Overall, the first-order feedback of the negative density dependence accounted for 20% of the variation in the annual population change rate and effectively regulated the population change rate of *H. armigera* to fluctuate around zero before and after the 1992 outbreak.

Density dependence can be reflected in the behavior and physiology of focal species (Turchin 1999), mainly due to intra- and interspecific competition and predation (Hixon and Johnson 2001). Cannibalism is common in *H. armigera* especially when under poor nutrition or high larval rearing density (Kakimoto et al. 2003), trimming population size and disrupting demographic structures. Pathogenic diseases and parasitism from natural enemies are also important factors to enhance the larval mortality of *H. armigera* especially when the larval density is high (Tschinkel 1981). Moreover, the population of *H. armigera* can easily revive from a low density due to polyphagy, high mobility, high fecundity, and facultative diapause. These behavioral and physiological characteristics could explain the relatively strong density dependence in the overwintering, second, and third generations. The reason that no significant density dependence was detected in the first generation (Fig.1B, A_1_) could be related to the long-distance migratory movement of adult *H. armigera* in the first generation from other regions in early June (Fig.1B).

### Climate change

Climate can not only regulate population dynamics (Coulson et al. 2000; Knape and de Valpine 2011) but also affect the strength of density dependence (Barbraud and Weimerskirch 2003). Time-series analyses have suggested that climatic variation can strongly affect the population change rate, after accounting for the role of density dependence and demographic stochasticity (Grotan et al. 2008). Rising temperature has increased the population growth rate of many insect herbivores (Veteli et al. 2002). Temperature also plays a crucial role in the development and survival of cotton bollworms (Wu and Guo 2005). Moderate temperature is crucial for hatching the eggs of the third generation during mid July and early August (Wu et al. 2008), while the optimal temperature for adult breeding ranges from 25 to 30°C (Wu and Guo 2005). Warm weather can enhance the breeding success and promote the development of larva, thus increasing the population density. Indeed, our results demonstrated that the relatively high temperature in the summer season can enhance population growth of cotton bollworms in the third generation (Fig.3I, Table1). Moreover, our results showed that the strength of density dependence was negatively correlated with summer temperature in the third generation; that is, rising temperature weakens the strength of density dependence. Higher temperature can lead to an extended growing season and thus an increase in plant biomass (Shaver et al. 2000; Veteli et al. 2002; Fang et al. 2003, 2004; Peng et al. 2011). The increase in plant biomass means more food resources for the polyphagous *H. armigera* and thus an elevated carrying capacity which limits cannibalism and competition. Consequently, rising temperature weakens the strength of density dependence. The elevated carrying capacity supported the population of *H. armigera* to fluctuate at a higher density level after 1992. Indeed, temperature-driven changes in system stability can account for the recurrent outbreaks of many other pest insects such as tea tortrix *Adoxophyes honmai* (Nelson et al. 2013).

Change in rainfall pattern has become evident in the region toward the end of last century (Prendergast 2008). Previous studies have shown that the outbreak of oriental migratory locust *Locusta migratoria manilensis* is associated with the floods and droughts in the lower Yangtze River (Stige et al. 2007). Earlier rainy season can drastically inhibit the population development of *H. armigera*, while the population usually reaches the outbreak status in later generations of the year during the drought season (Wu and Guo 2005). Our results showed an opposite effect of precipitation on the population change rate in the overwintering generation versus in the third generation of the summer season (Fig.3D vs. Fig.3J, Table1). The precipitation also affected the strength of density dependence in the third generation, with a significant positive interaction between density dependence and precipitation. The decreasing precipitation weakened the strength of density dependence, facilitating the switch of population equilibrium of *H. armigera* to the higher density level after 1992.

### Agricultural intensification

Many insect herbivores are mainly regulated by bottom-up processes, primarily by food availability (Steffan-Dewenter and Schiele 2008; Schowalter 2011). Increasing quality and quantity of food recourses through agricultural activity can promote pest population growth (Schowalter 2011). Agricultural production in the study area aims at increasing yield, reflected by the rising irrigation area that is closely correlated with the total crop yield. Our result showed that rising irrigation area enhanced the annual and two generational population change rates of the cotton bollworm (Fig.3E and G, Table1). Agricultural activity also affected the strength of density dependence in the second generation population, with a significant negative interaction also detected between the density dependence and irrigation area. Agricultural intensification can directly supply more food resources to the polyphagous *H. armigera*, elevating the carrying capacity and weakening the strength of density dependence. Agricultural intensification provided yet another trigger, together with climate change, that unleashed the pest population of *H. armigera* from the strong density-dependent regulation.

## Conclusion

Density dependence, climatic change, human activity, and demographic stochasticity, as well as their often complex interactions, are important population regulators (Bjornstad and Grenfell 2001). Quantitative time-series analysis in this study provided strong evidence on the mechanism of population regulation in the cotton bollworm. Although negative density dependence have effectively regulated the population change rate to close to zero, the exogenous factors of climate change and agricultural intensification exacerbated the weakening of negative density dependence and triggered outbreak in the 1992 of *H. armigera* in the region. Rising temperature, decreasing rainfall, and intensifying agricultural production weakened the strength of density dependence which allowed the population of *H. armigera* to fluctuate at a higher density level with larger amplitudes after the 1992 outbreak. Our results suggest that climate change and agricultural intensification in this Anthropocene could potentially induce the outbreaks of many pest insects through weakening the density-dependent population regulation. Insect pests that are tightly regulated by density-dependent mechanisms should be put on a watch list for potential threats to the provision of agro-ecosystem service and regional food security.

## Supporting Information

**Figure S1.** Partial autocorrelation functions (PACF) for the cotton bollworm in time series. **Figure S2.** Variation partitioning of population change rates (R-functions) for cotton bollworms explained by density. **Figure S3.** Residual diagnostics for annual model and for generational models. **Figure S4.** Yearly temperature, precipitation, and irrigation area at Raoyang county scale during 1980–2010. **Figure S5.** Introduction of time moving-window method. **Figure S6.** Moving-window analyses for the relationship between strength of density dependence and the precipitation for the overwinter generation. **Figure S7.** Moving-window analyses for the relationship between strength of density dependence and irrigation area for the second generation. **Figure S8.** Moving-window analyses for the relationship between strength of density dependence and the temperature for the third generation. **Figure S9.** Moving-window analyses for the relationship between strength of density dependence and the precipitation for the third generation. **Figure S10.** Moving-window analyses for the relationship between effects of the temperature on the third generation R-function and the precipitation.

**Table S1.** Dependent and explanatory variables in Eqn. (1) to Eqn. (5).

**Table S2.** Best-fitting main-effect population dynamic models (GAM and LM).

**Table S3.** Main-effect nonlinear candidate models (GAM).

**Table S4.** Main-effect linear candidate models (LM).

**Table S5.** Candidate models with interactions.
