# Supplementary material for: Weakening density dependence from climate change and agricultural intensification triggers pest outbreaks: a 37-year observation of cotton bollworms
Source: Ecol Evol. 2014 Aug 12;4(17):3362–74. doi: 10.1002/ece3.1190 (PMC4228611; doi:10.1002/ece3.1190)
Supplement: Supplementary file 1 [file ece30004-3362-sd1.doc]

**
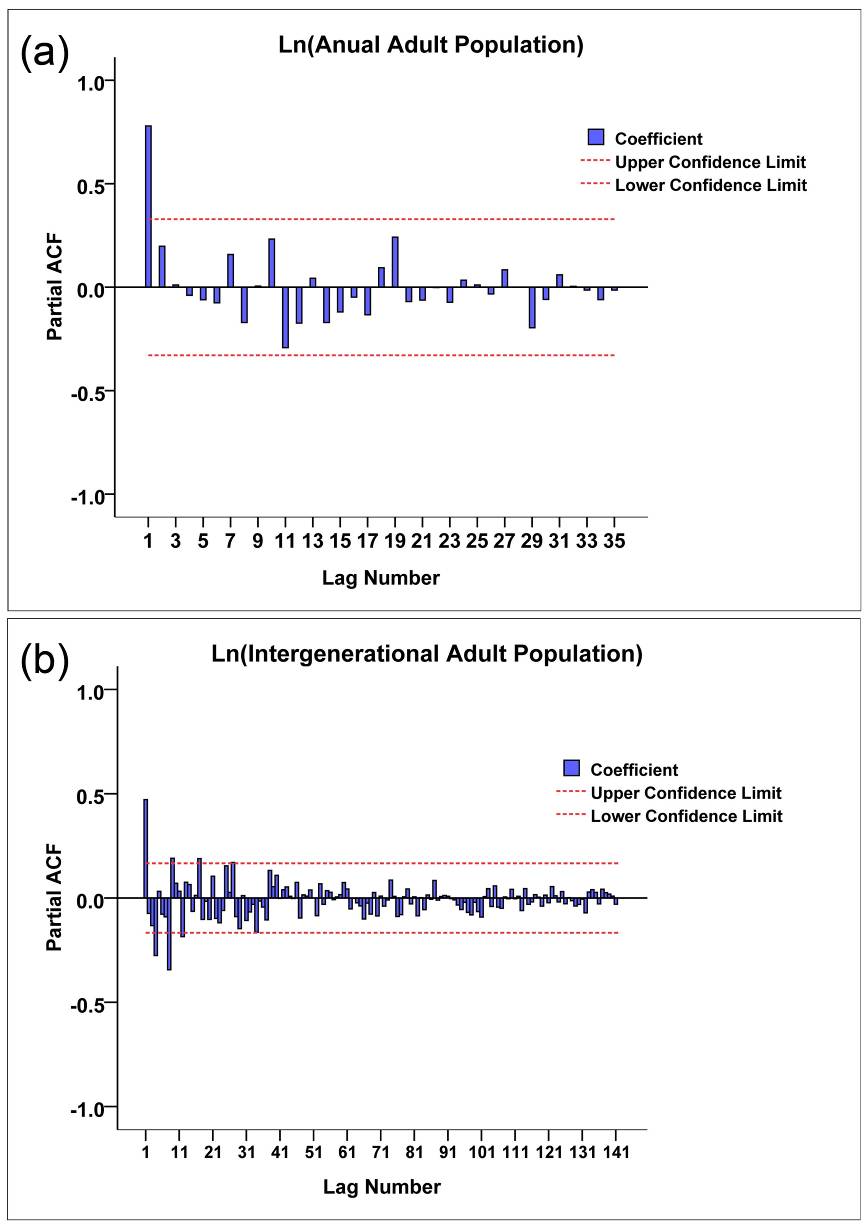
**

**Fig. S1. Partial autocorrelation functions (*PACF*) for the cotton bollworm in time series.** *PACF* of the annual population dynamic (a) and the generational population dynamic (b). The bars indicate the values of *PACF* at the corresponding lags. The bars that are beyond the upper or lower confidence limit are statistically significant in the time series.


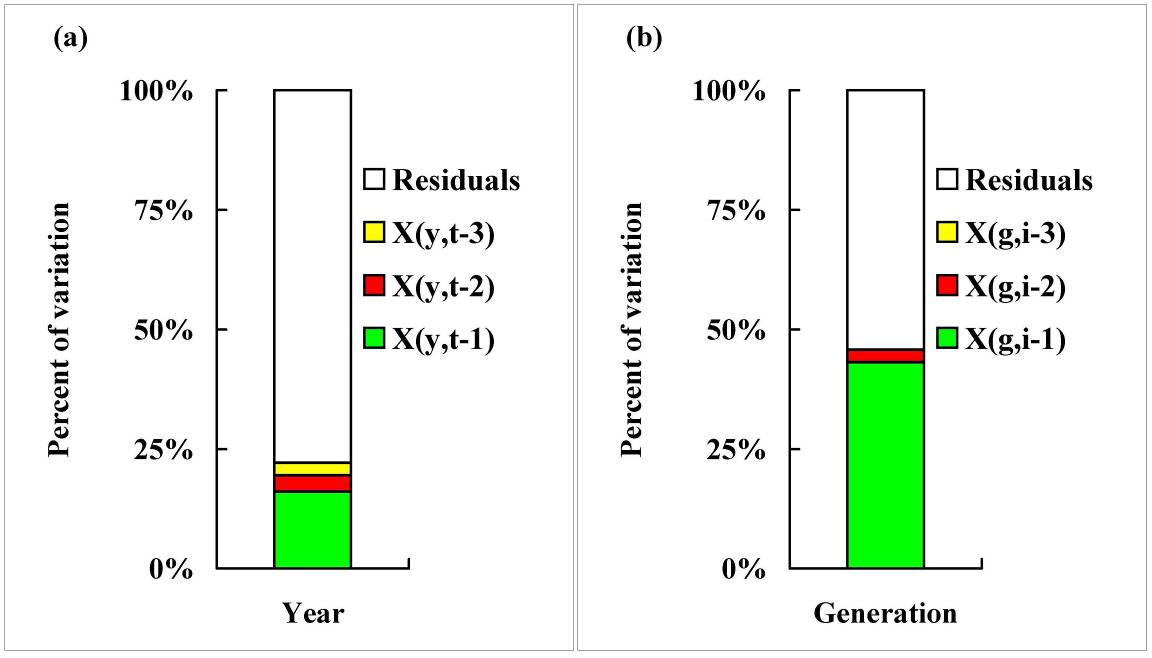


**Fig. S2. Variation partitioning of population change rates (*R*-functions) for cotton bollworm explained by density.** Population from horizontal direction consist of the generational and the yearly abundance. Vertical direction is percentage of variations. The variations were partitioned according to yearly densities of first-order (Xy,t-1), second-order (Xy,t-2) and third-order (Xy,t-3)(a). The variations were partitioned according to generational densities of first-order (Xg,t-1), second-order (Xg,t-2) and third-order (Xg,t-3)(b).


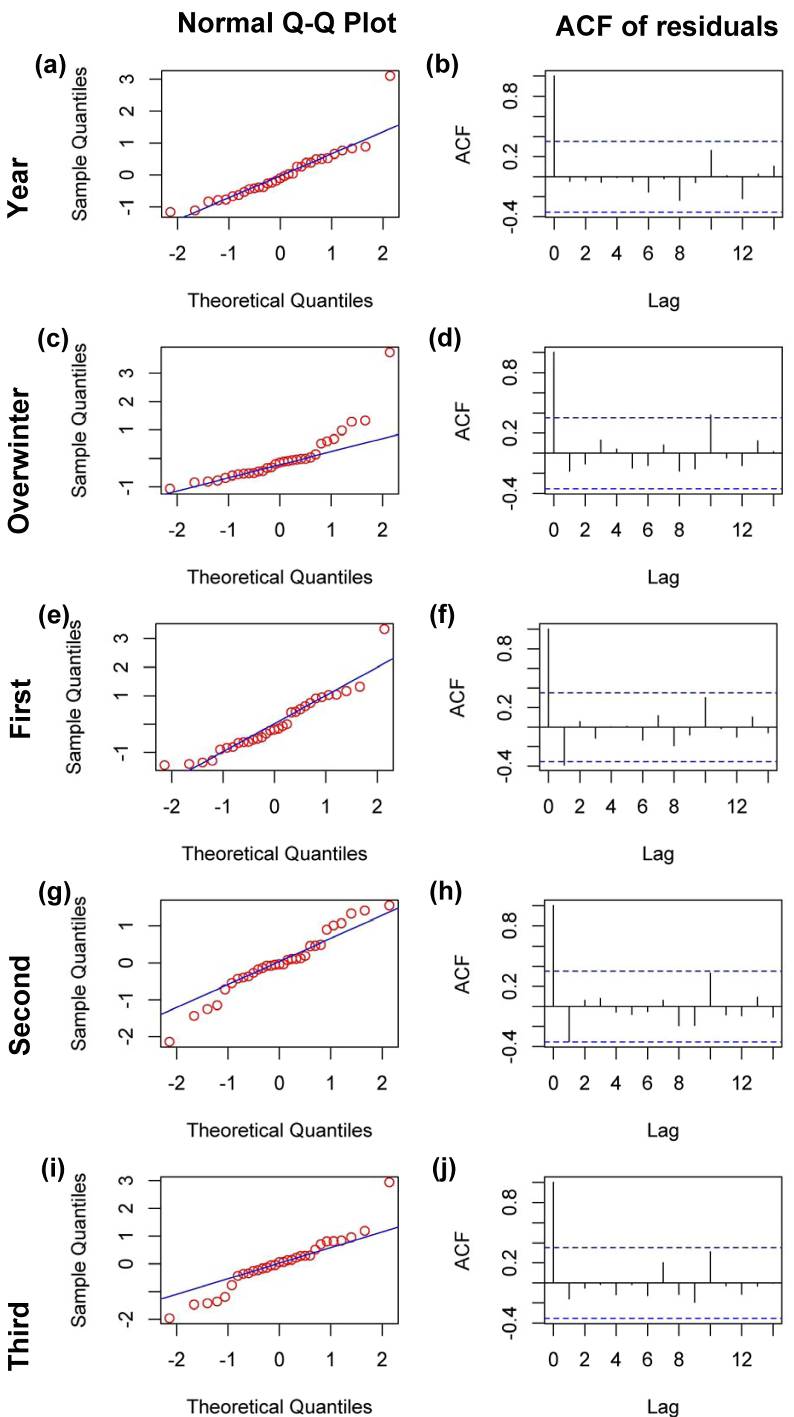


**Fig. S3. Residual diagnostics for annual model (a, b), and for generational models in the overwinter (c, d) , first (e, f), second (g, h)and third generation (i, j).** Residuals are approximately normally distributed (the quantile plot of residuals forms a nearly straight line). Autocorrelation function of residuals (ACF) reveals no significant autocorrelation.

**
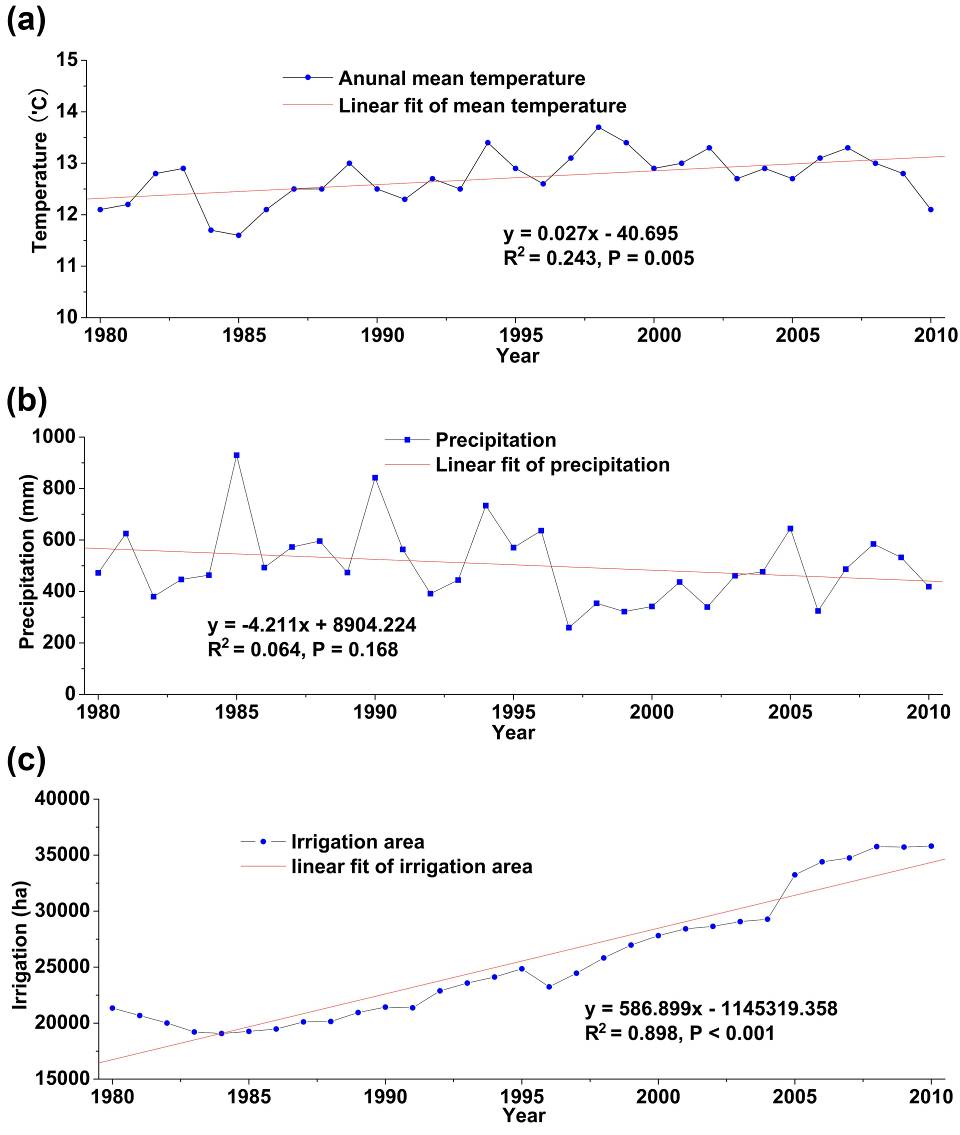
**

**Fig. S4. Yearly temperature (a), precipitation (b) and irrigation area (c) at Raoyang county scale during 1980 to 2010**

**
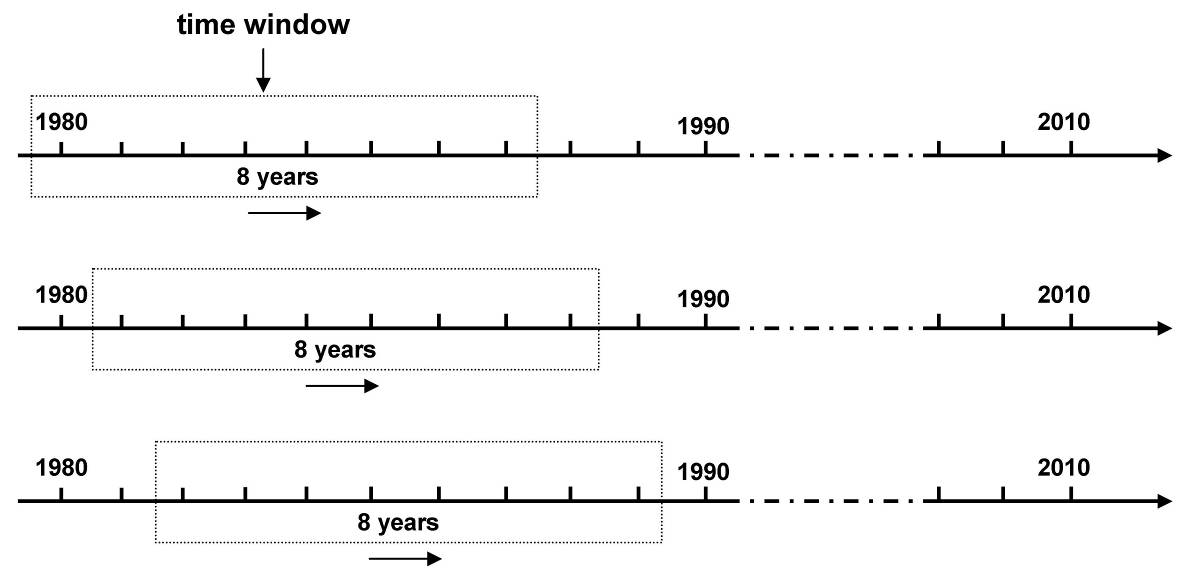
**

**Fig. S5. Time moving-window method in analysis of this paper.** The time windows we adopted in this study are 7-12 years, respectively.

**
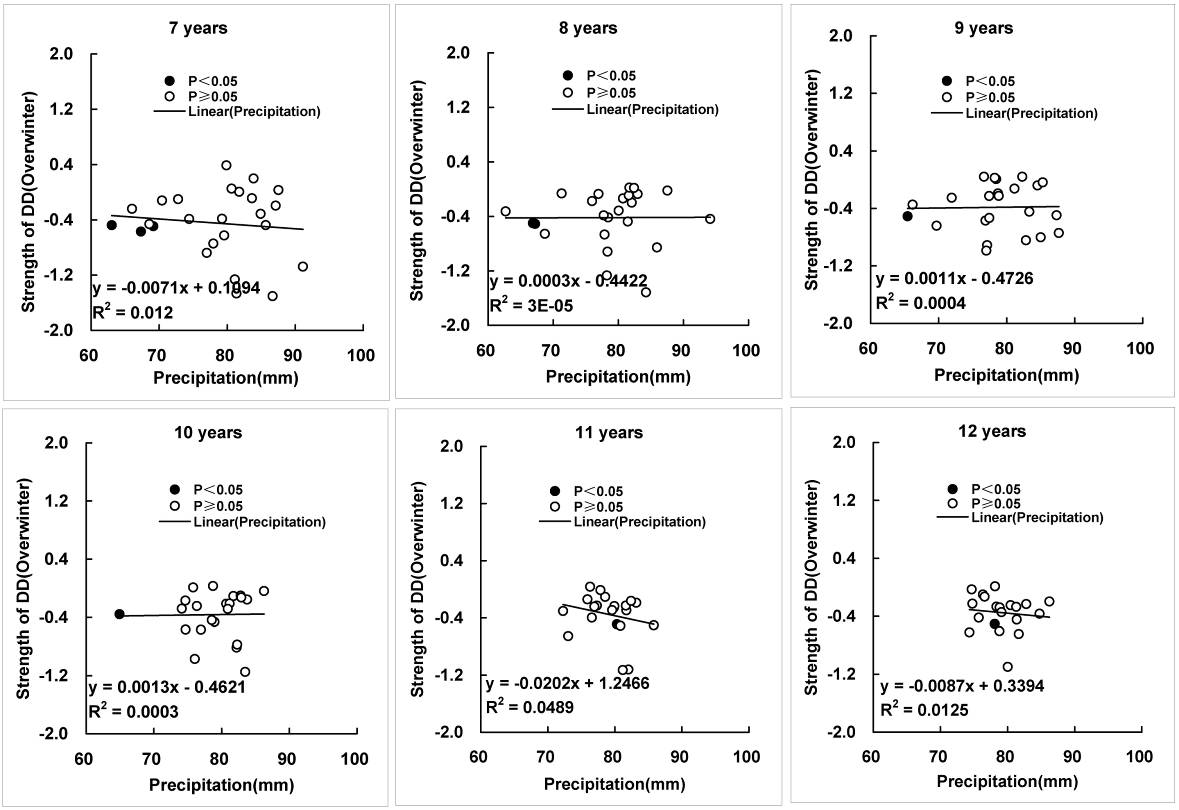
**

**Fig. S6 Moving-window analyses for the relationship between strength of DD for the overwinter generation and the precipitation during period of the overwinter generation (Precipitation_O,t_).** The values of strength of DD were from coefficient b in *R*_O,t_ = a *+* b × *X*_T,t-1_ (solid dots: p < 0.05, hollow dots: p ≥ 0.05; similarly hereinafter). The time windows we adopted in our study are 7-12 years, respectively. The titles refer to different time windows (similarly hereinafter).

**
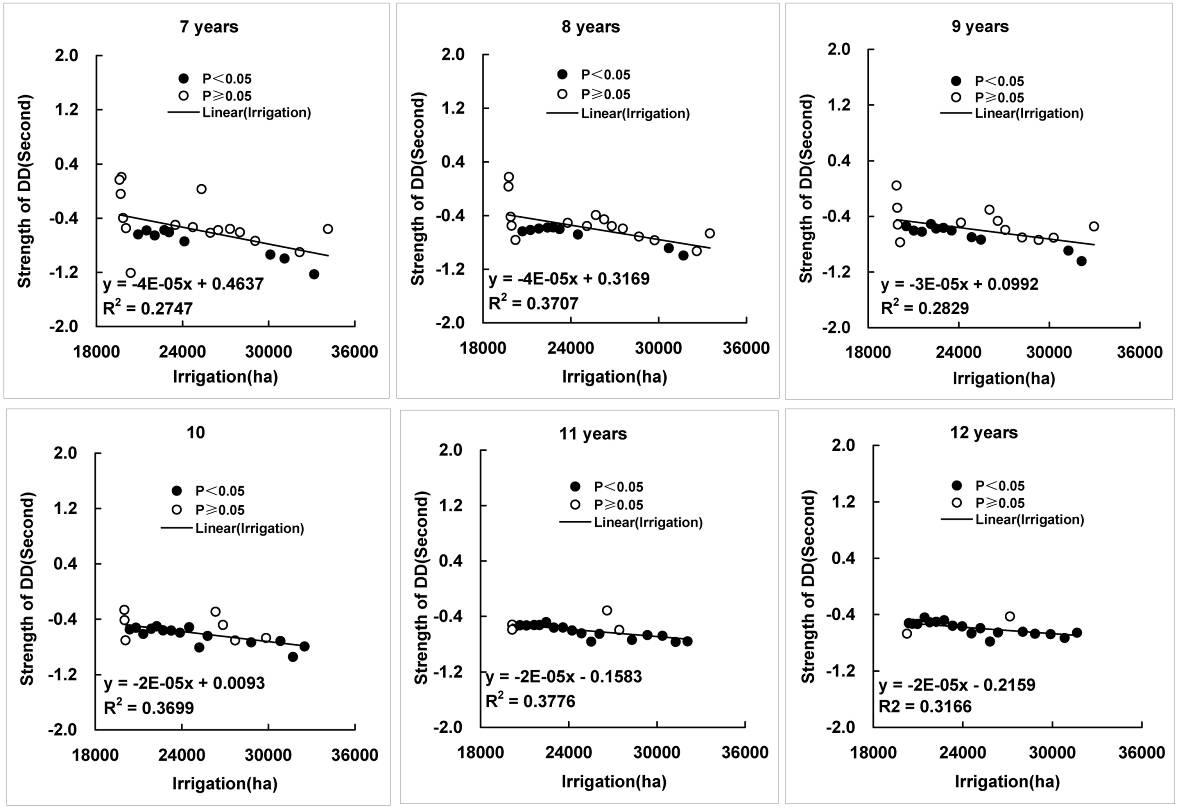
**

**Fig. S7 Moving-window analyses for the relationship between strength of DD for the second generation and irrigation area (Irrigation_Y,t_).** The values of strength of DD were from coefficient b in *R*_S,t_ = a *+* b × *X*_F,t_ (solid dots: p < 0.05, hollow dots: p ≥ 0.05; similarly hereinafter). The time windows we adopted in our study are 7-12 years, respectively. The titles refer to different time windows (similarly hereinafter).


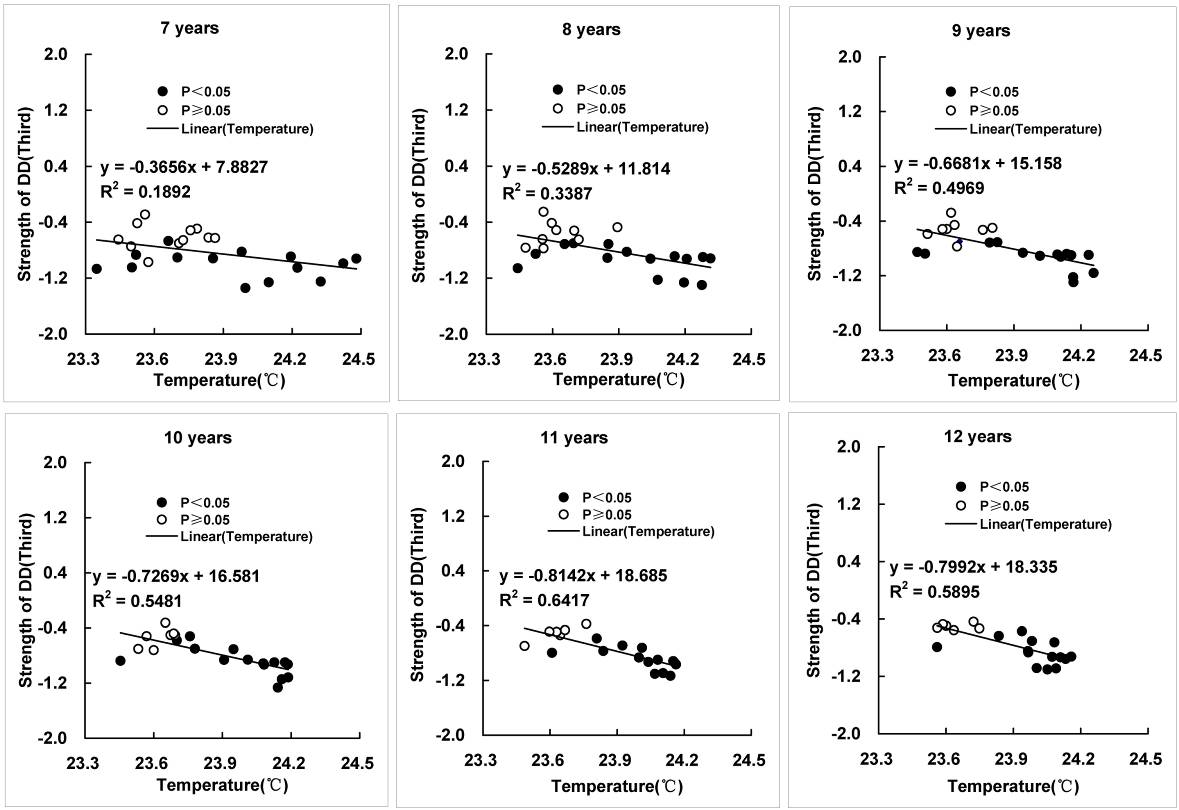


**Fig. S8 Moving-window analyses for the relationship between strength of DD for the third generation and the temperature during period of the third generation (Temperature_T,t_).** The values of strength of DD were from coefficient b in *R*_T,t_ = a *+* b × *X*_S,t_ (solid dots: p < 0.05, hollow dots: p ≥ 0.05; similarly hereinafter). The time windows we adopted in our study are 7-12 years, respectively. The titles refer to different time windows (similarly hereinafter).

**
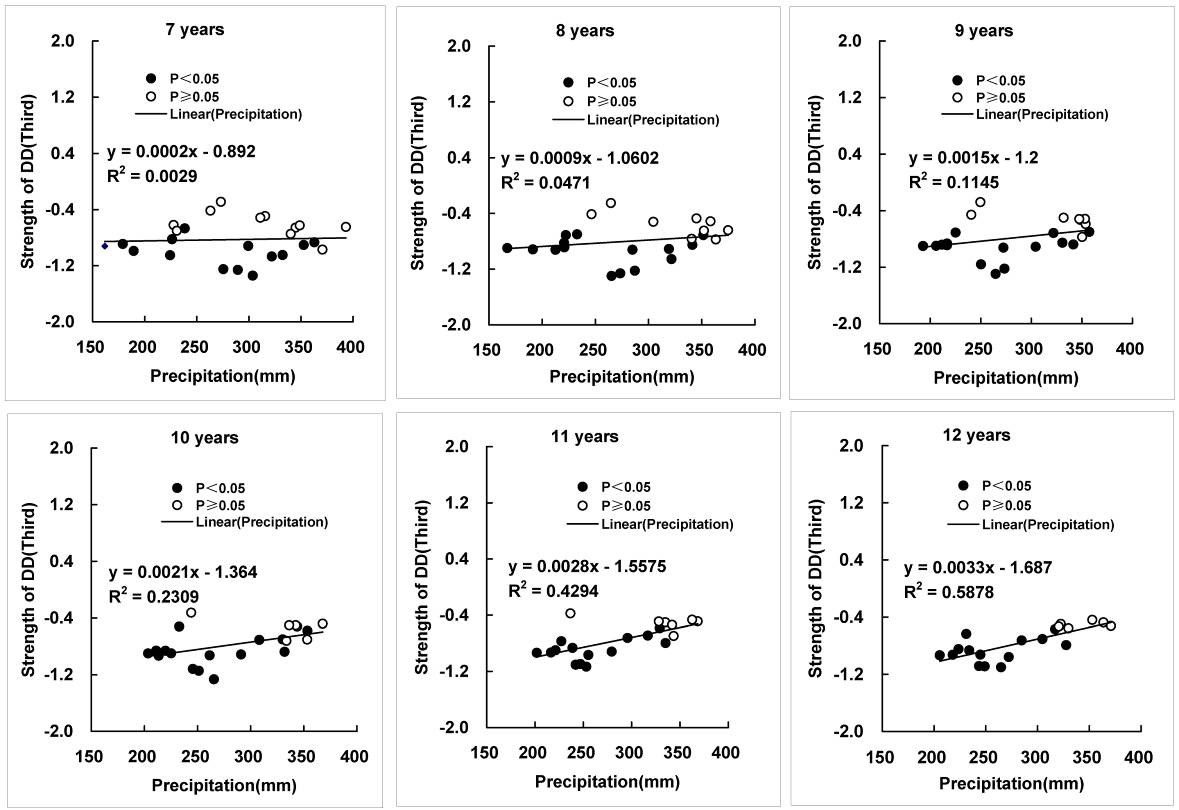
**

**Fig. S9 Moving-window analyses for the relationship between strength of DD for the third generation and the precipitation during period of the third generation (Precipitation_T,t_).** The values of strength of DD were from coefficient b in *R*_T,t_ = a *+* b × *X*_S,t_ (solid dots: p < 0.05, hollow dots: p ≥ 0.05; similarly hereinafter). The time windows we adopted in our study are 7-12 years, respectively. The titles refer to different time windows (similarly hereinafter).


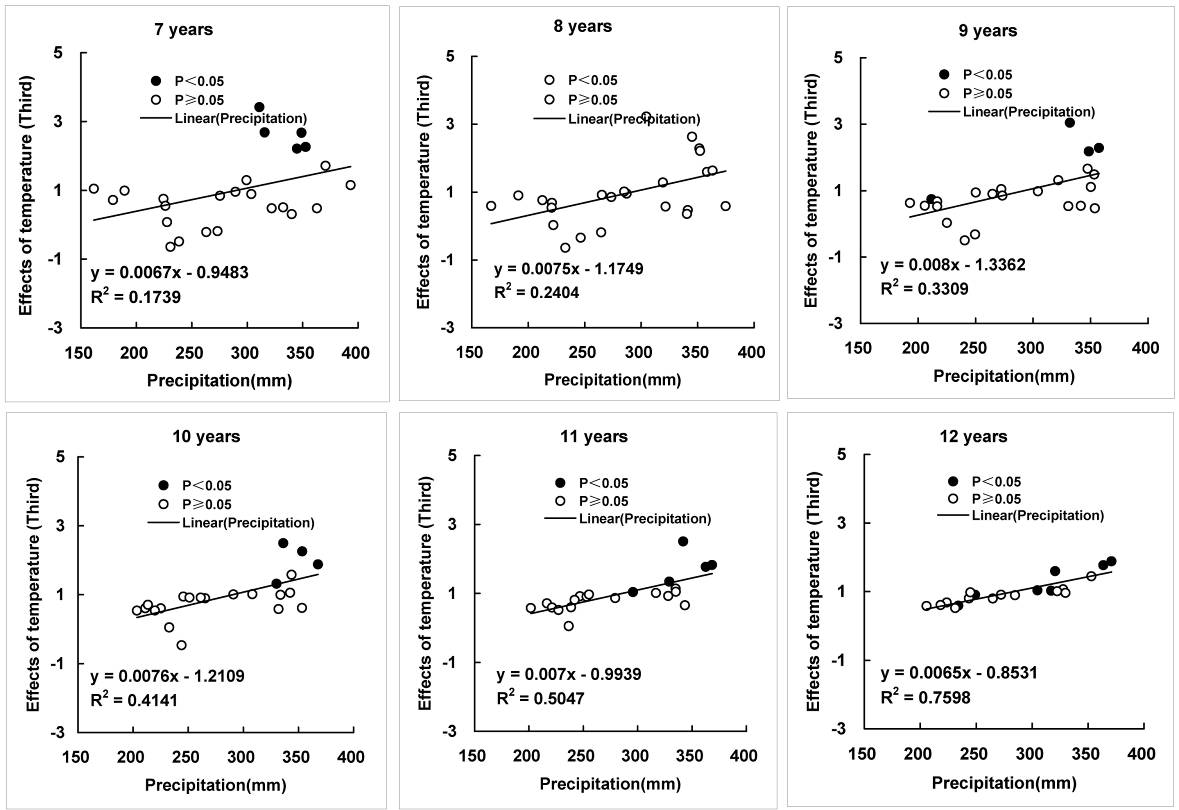


**Fig. S10** **Moving-window analyses for the relationship between effects of the temperature (Temperature_T,t_) on the third generation R-function and the precipitation (Precipitation_T,t_).** The values of effects of temperature were from coefficient b in *R*_T,t_ = a *+* b × Temperature_T,t_ (solid dots: p < 0.05, hollow dots: p ≥ 0.05; similarly hereinafter). The time windows we adopted in our study are 7-12 years, respectively. The titles refer to different time windows (similarly hereinafter).
