# Supplementary material for: Weakening density dependence from climate change and agricultural intensification triggers pest outbreaks: a 37-year observation of cotton bollworms
Source: Ecol Evol. 2014 Aug 12;4(17):3362–74. doi: 10.1002/ece3.1190 (PMC4228611; doi:10.1002/ece3.1190)
Supplement: Supplementary file 2 — Table S1. Dependent and explanatory variables in Eqn. (1) to Eqn. (5). [file ece30004-3362-sd2.doc]

**Table S1 Dependent and explanatory variables, selected predictors, divided into categories and subcategories that potentially affected population change rate of cotton bollworm in eqn 1 to eqn 5.**

| **Dependent variable** | **Explanatory variables** | | | |
| --- | --- | --- | --- | --- |
| **Population change rate** | **Factors** | **Categories** | **Subcategories** | **Predictors** |
| **Annual model** | | | | |
| All the year (*R*_Y,t_) | Endogenous density-dependent | Density | Number of adult | Natural logarithmic abundance in last year of the whole (*X*_Y,t-1_) |
|  |  |  |  |  |
|  | Exogenous density-independent | Climate | Temperature | Annual mean temperature (*T*_Y,t_) |
|  |  |  | Precipitation | Annual total rainfall (*P*_Y,t_) |
|  |  | Human activity^a^ | Irrigation area | Annual total irrigation (*I*_Y,t_) |
|  | | | | |
| **Generational models** | | | | |
| Overwinter generation (*R*_O,t_) | Endogenous density-dependent | Density | Number of adult | Natural logarithmic abundance in the third generation of last year (*X*_T,t-1_) |
|  |  |  |  |  |
|  |  |  |  |  |
|  | Exogenous density-independent | Climate | Temperature | Mean, minimum or maximum temperature, or accumulative total temperature below zero during the overwinter generation (*T*mean_O,t_, *T*min_O,t_, *T*man_O,t_ and *T*total_O,t_ ) |
|  |  |  | Precipitation | Total rainfall during the overwinter generation (*P*_O,t_) |
|  |  | Human activity | Irrigation area | Total irrigation area during the overwinter generation (*I*_O,t_) |
| First generation (*R*_F,t_) | Endogenous density-dependent | Density | Number of adult | Natural logarithmic abundance in the overwinter generation (*X*_O,t_) |
|  | Exogenous density-independent | Climate | Temperature | Mean temperature during period of the first generation (*T*_F,t_) |
|  |  |  | Precipitation | Total rainfall during the first generation (*P*_F,t_) |
|  |  | Human activity | Irrigation area | Total irrigation area during the first generation (*I*_F,t_) |
| Second generation (*R*_S,t_) | Endogenous density-dependent | Density | Number of adult | Natural logarithmic abundance in the first generation (*X*_F,t_) |
|  |  |  |  |  |
|  | Exogenous density-independent | Climate | Temperature | Mean temperature during the second generation (*T*_S,t_) |
|  |  |  | Precipitation | Total rainfall during the second generation (*P*_S,t_) |
|  |  | Human activity | Irrigation area | Total irrigation area during the second generation (*I*_S,t_) |
| Third generation (*R*_T,t_) | Endogenous density-dependent | Density | Number of adult | Natural logarithmic abundance in the second generation (*X*_S,t_) |
|  |  |  |  |  |
|  | Exogenous density-independent | Climate | Temperature | Mean temperature during the third generation (*T*_T,t_) |
|  |  |  | Precipitation | Total rainfall during the third generation (*P*_T,t_) |
|  |  | Human activity | Irrigation area | Total irrigation area during the third generation (*I*_T,t_) |

^a^ Predictors of yield of crops, quantity of agricultural machines, mechanomotive force of agricultural machines, ploughing area are collinearity with irrigation area, thus only irrigation area is as the explanatory variables of human activity in analysis models.
