# Supplementary material for: Weakening density dependence from climate change and agricultural intensification triggers pest outbreaks: a 37-year observation of cotton bollworms
Source: Ecol Evol. 2014 Aug 12;4(17):3362–74. doi: 10.1002/ece3.1190 (PMC4228611; doi:10.1002/ece3.1190)
Supplement: Supplementary file 3 — Table S2. Best-fitting main-effect population dynamic models (GAM and LM). [file ece30004-3362-sd3.doc]

**Table S2. Best-fitting main-effect population dynamic models (GAM and LM) fitted for adult *H. armigera.***

| **Population of generation** | **Models** | **Dev.expl** | **GCV** |
| --- | --- | --- | --- |
|  | **Generalized additive models** |  |  |
| the whole year | *R*_Y, t_ = *f*_1_(*X*_Y_*,*_t-1_) + *h*_1_(Irrigation_Y,t_) | 40.5% | 0.895 |
| the overwinter | *R*_O, t_ = *f*_2_(*X*_T_*,*_t-1_) + *g*_4_(Precipitation_O,t_) | 36.0% | 1.024 |
| the first | *R*_F, t_ = *h*_3_(Irrigation_Y,t_) | 25.7% | 1.303 |
| the second | *R*_S,t_ = *f*_4_(*X*_F_*,*_t_) + *h*_4_(Irrigation_Y,t_) | 54.1% | 0.930 |
| the third | *R*_T,t_ = *f*_5_(*X*_S_*,*_t_) + *g*_9_ (Temperature_T,t_)+*g*_10_(Precipitation_T,t_) | 65.0% | 0.696 |
|  | **Linear regression models** |  |  |
| the whole year | *R*_Y, t_ = 2.3860-0.2790*X*_Y_*,*_t-1_ | 15.7% | 1.049 |
| the overwinter | *R*_O, t_ = -0.204094-0.352788*X*_T_*,*_t-1_*-*0.01343Precipitation_O,t_ | 36.0% | 1.024 |
| the first | *R*_F, t_ = 1.3340+0.00007735Irrigation_Y,t_ | 12.7% | 1.440 |
| the second | *R*_S,t_ = 0.4504-0.4840*X*_F_*,*_t_ + 0.0001117Irrigation_Y,t_ | 39.8% | 1.134 |
| the third | *R*_T,t_ = -18.356884*-*0.435659*X*_S_*,*_t_+ 0.888708Temperature_T,t_+0.002787Precipitation_T,t_ | 65.0% | 0.696 |

Models were selected using the generalized cross-validation value (GCV) on the condition that all variables must be statistically significant (P < 0.05). Parameter values are defined as follows. *R*_Y,t_, *R*_O, t_, *R*_F, t_, *R*_S,t_ and *R*_T,t_ are the adult population change rate in the whole year, the overwinter, the first, the second and the third generations, respectively. *X*_Y_*,*_t-1_ is natural log-transformed annual density of *H. armigera* in year *t-1*. Ln-transformed generational abundances of *H. armigera* in the overwinter, first, second and third generation population of year t are *X*_O,t_, *X*_F,t_, *X*_S,t_ and third *X*_T,t_, respectively. Annual irrigation area in year t is Irrigation_Y,t_. The temperature and precipitation variables used in the following models are generational variables covering the respective generational population. The mean temperature in the period of the whole year, and the overwinter, the first, the second and the third generations are Temperature_Y,t_, Temperature_O,t_, Temperature_F,t_, Temperature_S,t_, and Temperature_T,t_. The precipitation in the period of the whole year, the overwinter, the first, the second and the third generations are Precipitation_Y,t_, Precipitation_O,t_, Precipitation_F,t_, Precipitation_S,t_ and Precipitation_T,t_. Beside, the mean, max and min temperature in the period of the overwinter generation are Tmean, Tmax and Tmin. T total is the accumulative total subzero temperature (accumulative total temperature in the period of the overwinter generation when it is below zero). Dev.expl is proportion of deviance explained by the models.
