# Supplementary material for: Weakening density dependence from climate change and agricultural intensification triggers pest outbreaks: a 37-year observation of cotton bollworms
Source: Ecol Evol. 2014 Aug 12;4(17):3362–74. doi: 10.1002/ece3.1190 (PMC4228611; doi:10.1002/ece3.1190)
Supplement: Supplementary file 4 — Table S3. Main-effect nonlinear candidate models (GAM). [file ece30004-3362-sd4.doc]

**Table S3** **Main-effect nonlinear candidate models.** Main-effect population dynamic models (GAM) fitted for adult cotton bollworm population of the whole year generation, the overwinter generation, the first generation, the second generation, the third generation.

|  | **the whole year generation** | | | | | | | |
| --- | --- | --- | --- | --- | --- | --- | --- | --- |
| **No.** | **Item** | DF | Estimate | t value | F value | p value | Dev.expl | GCV |
| **1** | **Model** | 2 |  |  |  |  | 15.70% | 1.049 |
|  | **Intercept** | 1 | 0.04965 | 0.279 |  | 0.782 |  |  |
|  | ***X*_Y,t-1_** | 1 |  |  | 5.385 | 0.027 |  |  |
| **2** | **Model** | 2 |  |  |  |  | 0.45% | 1.238 |
|  | **Intercept** | 1 | 0.04965 | 0.257 |  | 0.799 |  |  |
|  | **Temperature_Y,t_** | 1 |  |  | 0.131 | 0.720 |  |  |
| **3** | **Model** | 2.75 |  |  |  |  | 10.90% | 1.167 |
|  | **Intercept** | 1 | 0.04965 | 0.268 |  | 0.791 |  |  |
|  | **Precipitation_Y,t_** | 1.75 |  |  | 1.136 | 0.335 |  |  |
| **4** | **Model** | 2 |  |  |  |  | 1.84% | 1.221 |
|  | **Intercept** | 1 | 0.04965 | 0.259 |  | 0.798 |  |  |
|  | **Irrigation_Y,t_** | 1 |  |  | 0.542 | 0.467 |  |  |
| **5** | **Model** | 3 |  |  |  |  | 20.70% | 1.059 |
|  | **Intercept** | 1 | 0.04965 | 0.283 |  | 0.779 |  |  |
|  | ***X*_Y,t-1_** | 1 |  |  | 7.134 | 0.012 |  |  |
|  | **Temperature_Y,t_** | 1 |  |  | 1.766 | 0.195 |  |  |
| **6** | **Model** | 3.78 |  |  |  |  | 23.30% | 1.083 |
|  | **Intercept** | 1 | 0.04965 | 0.284 |  | 0.779 |  |  |
|  | ***X*_Y,t-1_** | 1 |  |  | 4.453 | 0.044 |  |  |
|  | **Precipitation_Y,t_** | 1.78 |  |  | 0.944 | 0.403 |  |  |
| **7** | **Model** | 4.63 |  |  |  |  | 40.50% | 0.895 |
|  | **Intercept** | 1 | 0.04965 | 0.317 |  | 0.754 |  |  |
|  | ***X*_Y,t-1_** | 1 |  |  | 15.725 | < 0.001 |  |  |
|  | **Irrigation_Y,t_** | 2.63 |  |  | 3.345 | 0.035 |  |  |
| **8** | **Model** | 3.76 |  |  |  |  | 11.40% | 1.249 |
|  | **Intercept** | 1 | 0.04965 | 0.264 |  | 0.794 |  |  |
|  | **Temperature_Y,t_** | 1 |  |  | 0.039 | 0.845 |  |  |
|  | **Precipitation_Y,t_** | 1.76 |  |  | 1.232 | 0.306 |  |  |
| **9** | **Model** | 3 |  |  |  |  | 1.85% | 1.310 |
|  | **Intercept** | 1 | 0.04965 | 0.254 |  | 0.801 |  |  |
|  | **Temperature_Y,t_** | 1 |  |  | 0.004 | 0.950 |  |  |
|  | **Irrigation_Y,t_** | 1 |  |  | 0.399 | 0.533 |  |  |
| **10** | **Model** | 3.72 |  |  |  |  | 11.50% | 1.244 |
|  | **Intercept** | 1 | 0.04965 | 0.264 |  | 0.794 |  |  |
|  | **Precipitation_Y,t_** | 1.72 |  |  | 1.071 | 0.357 |  |  |
|  | **Irrigation_Y,t_** | 1 |  |  | 0.267 | 0.610 |  |  |
| **11** | **Model** | 5.22 |  |  |  |  | 30.00% | 1.101 |
|  | **Intercept** | 1 | 0.04965 | 0.289 |  | 0.775 |  |  |
|  | ***X*_Y,t-1_** | 1 |  |  | 6.367 | 0.018 |  |  |
|  | **Temperature_Y,t_** | 1.47 |  |  | 1.461 | 0.242 |  |  |
|  | **Precipitation_Y,t_** | 1.74 |  |  | 1.010 | 0.379 |  |  |
| **12** | **Model** | 5.58 |  |  |  |  | 40.60% | 0.961 |
|  | **Intercept** | 1 | 0.04965 | 0.311 |  | 0.758 |  |  |
|  | ***X*_Y,t-1_** | 1 |  |  | 15.094 | 0.001 |  |  |
|  | **Temperature_Y,t_** | 1 |  |  | 0.228 | 0.637 |  |  |
|  | **Irrigation_Y,t_** | 2.58 |  |  | 2.538 | 0.081 |  |  |
| **13** | **Model** | 5.57 |  |  |  |  | 41.30% | 0.949 |
|  | **Intercept** | 1 | 0.04965 | 0.313 |  | 0.757 |  |  |
|  | ***X*_Y,t-1_** | 1 |  |  | 14.959 | 0.001 |  |  |
|  | **Precipitation_Y,t_** | 1 |  |  | 0.476 | 0.496 |  |  |
|  | **Irrigation_Y,t_** | 2.57 |  |  | 3.382 | 0.035 |  |  |
| **14** | **Model** | 4.71 |  |  |  |  | 11.60% | 1.337 |
|  | **Intercept** | 1 | 0.04965 | 0.260 |  | 0.797 |  |  |
|  | **Temperature_Y,t_** | 1 |  |  | < 0.001 | 0.997 |  |  |
|  | **Precipitation_Y,t_** | 1.71 |  |  | 1.051 | 0.364 |  |  |
|  | **Irrigation_Y,t_** | 1 |  |  | 0.222 | 0.641 |  |  |
| **15** | **Model** | 6.5 |  |  |  |  | 41.90% | 1.013 |
|  | **Intercept** | 1 | 0.04965 | 0.309 |  | 0.760 |  |  |
|  | ***X*_Y,t-1_** | 1 |  |  | 14.802 | 0.001 |  |  |
|  | **Temperature_Y,t_** | 1 |  |  | 0.507 | 0.483 |  |  |
|  | **Precipitation_Y,t_** | 1 |  |  | 0.728 | 0.402 |  |  |
|  | **Irrigation_Y,t_** | 2.5 |  |  | 2.569 | 0.080 |  |  |
|  |  |  |  |  |  |  |  |  |
|  | **the overwinter generation** | | | | | | | |
| **No.** | **Item** | DF | Estimate | t value | F value | p value | Dev.expl | GCV |
| **1** | **Model** | 2 |  |  |  |  | 19.20% | 1.204 |
|  | **Intercept** | 1 | -3.83640 | -20.130 |  | < 0.001 |  |  |
|  | ***X*_T,t-1_** | 1 |  |  | 6.906 | 0.014 |  |  |
| **2** | **Model** | 2 |  |  |  |  | 3.33% | 1.441 |
|  | **Intercept** | 1 | -3.83640 | -18.400 |  | < 0.001 |  |  |
|  | **Ttotal** | 1 |  |  | 0.997 | 0.326 |  |  |
| **3** | **Model** | 2 |  |  |  |  | 3.95% | 1.431 |
|  | **Intercept** | 1 | -3.83640 | -18.460 |  | < 0.001 |  |  |
|  | **Tmean** | 1 |  |  | 1.193 | 0.284 |  |  |
| **4** | **Model** | 2 |  |  |  |  | 0.05% | 1.489 |
|  | **Intercept** | 1 | -3.83600 | -18.090 |  | < 0.001 |  |  |
|  | **Tmax** | 1 |  |  | 0.015 | 0.902 |  |  |
| **5** | **Model** | 2 |  |  |  |  | 10.50% | 1.334 |
|  | **Intercept** | 1 | -3.83640 | -19.120 |  | < 0.001 |  |  |
|  | **Tmin** | 1 |  |  | 3.403 | 0.075 |  |  |
| **6** | **Model** | 2 |  |  |  |  | 19.10% | 1.206 |
|  | **Intercept** | 1 | -3.83640 | -20.110 |  | < 0.001 |  |  |
|  | **Precipitation_O,t_** | 1 |  |  | 6.849 | 0.014 |  |  |
| **7** | **Model** | 3.07 |  |  |  |  | 22.80% | 1.240 |
|  | **Intercept** | 1 | -3.83640 | -20.210 |  | < 0.001 |  |  |
|  | **Irrigation_Y,t_** | 2.07 |  |  | 2.479 | 0.092 |  |  |
| **8** | **Model** | 3 |  |  |  |  | 20.20% | 1.276 |
|  | **Intercept** | 1 | -3.83640 | -19.890 |  | < 0.001 |  |  |
|  | ***X*_T,t-1_** | 1 |  |  | 5.907 | 0.022 |  |  |
|  | **Ttotal** | 1 |  |  | 0.327 | 0.572 |  |  |
| **9** | **Model** | 3 |  |  |  |  | 19.80% | 1.282 |
|  | **Intercept** | 1 | -3.83640 | -19.850 |  | < 0.001 |  |  |
|  | ***X*_T,t-1_** | 1 |  |  | 5.538 | 0.026 |  |  |
|  | **Tmean** | 1 |  |  | 0.202 | 0.657 |  |  |
| **10** | **Model** | 3 |  |  |  |  | 23.10% | 1.229 |
|  | **Intercept** | 1 | -3.83640 | -20.270 |  | < 0.001 |  |  |
|  | ***X*_T,t-1_** | 1 |  |  | 8.409 | 0.007 |  |  |
|  | **Tmax** | 1 |  |  | 1.422 | 0.243 |  |  |
| **11** | **Model** | 3.02 |  |  |  |  | 19.60% | 1.287 |
|  | **Intercept** | 1 | -3.83640 | -19.820 |  | < 0.001 |  |  |
|  | ***X*_T,t-1_** | 1 |  |  | 3.138 | 0.087 |  |  |
|  | **Tmin** | 1.02 |  |  | 0.075 | 0.794 |  |  |
| **12** | **Model** | 3 |  |  |  |  | 36.00% | 1.024 |
|  | **Intercept** | 1 | -3.83640 | -22.210 |  | < 0.001 |  |  |
|  | ***X*_T,t-1_** | 1 |  |  | 7.372 | 0.011 |  |  |
|  | **Precipitation_O,t_** | 1 |  |  | 7.316 | 0.011 |  |  |
| **13** | **Model** | 3.69 |  |  |  |  | 25.10% | 1.259 |
|  | **Intercept** | 1 | -3.83640 | -20.280 |  | < 0.001 |  |  |
|  | ***X*_T,t-1_** | 1 |  |  | 2.461 | 0.128 |  |  |
|  | **Irrigation_Y,t_** | 1.69 |  |  | 0.680 | 0.519 |  |  |
| **14** | **Model** | 3 |  |  |  |  | 20.10% | 1.277 |
|  | **Intercept** | 1 | -3.83640 | -19.890 |  | < 0.001 |  |  |
|  | **Ttotal** | 1 |  |  | 0.356 | 0.556 |  |  |
|  | **Precipitation_O,t_** | 1 |  |  | 5.887 | 0.022 |  |  |
| **15** | **Model** | 4 |  |  |  |  | 22.90% | 1.326 |
|  | **Intercept** | 1 | -3.83600 | -19.880 |  | < 0.001 |  |  |
|  | **Ttotal** | 1 |  |  | 0.003 | 0.958 |  |  |
|  | **Irrigation_Y,t_** | 1 |  |  | 1.991 | 0.147 |  |  |
| **16** | **Model** | 4.03 |  |  |  |  | 36.40% | 1.097 |
|  | **Intercept** | 1 | -3.83640 | -21.870 |  | < 0.001 |  |  |
|  | **Precipitation_O,t_** | 1 |  |  | 6.175 | 0.019 |  |  |
|  | **Irrigation_Y,t_** | 2.03 |  |  | 2.202 | 0.120 |  |  |
| **17** | **Model** | 4 |  |  |  |  | 39.10% | 1.047 |
|  | **Intercept** | 1 | -3.83640 | -22.370 |  | < 0.001 |  |  |
|  | ***X*_T,t-1_** | 1 |  |  | 8.413 | 0.007 |  |  |
|  | **Ttotal** | 1 |  |  | 1.389 | 0.249 |  |  |
|  | **Precipitation_O,t_** | 1 |  |  | 8.393 | 0.007 |  |  |
| **18** | **Model** | 4.66 |  |  |  |  | 26.70% | 1.324 |
|  | **Intercept** | 1 | -3.83640 | -20.140 |  | < 0.001 |  |  |
|  | ***X*_T,t-1_** | 1 |  |  | 3.022 | 0.094 |  |  |
|  | **Ttotal** | 1 |  |  | 0.577 | 0.454 |  |  |
|  | **Irrigation_Y,t_** | 1.66 |  |  | 0.780 | 0.470 |  |  |
| **19** | **Model** | 4.41 |  |  |  |  | 38.70% | 1.086 |
|  | **Intercept** | 1 | -3.83640 | -22.130 |  | < 0.001 |  |  |
|  | ***X*_T,t-1_** | 1 |  |  | 3.888 | 0.059 |  |  |
|  | **Precipitation_O,t_** | 1 |  |  | 6.905 | 0.014 |  |  |
|  | **Irrigation_Y,t_** | 1.41 |  |  | 0.345 | 0.677 |  |  |
| **20** | **Model** | 4.98 |  |  |  |  | 37.20% | 1.163 |
|  | **Intercept** | 1 | -3.83640 | -21.620 |  | < 0.001 |  |  |
|  | **Ttotal** | 1 |  |  | 0.179 | 0.675 |  |  |
|  | **Precipitation_O,t_** | 1 |  |  | 6.198 | 0.019 |  |  |
|  | **Irrigation_Y,t_** | 1.98 |  |  | 2.141 | 0.129 |  |  |
| **21** | **Model** | 5.49 |  |  |  |  | 43.30% | 1.092 |
|  | **Intercept** | 1 | -3.83640 | -22.540 |  | < 0.001 |  |  |
|  | ***X*_T,t-1_** | 1 |  |  | 5.483 | 0.027 |  |  |
|  | **Ttotal** | 1 |  |  | 1.840 | 0.187 |  |  |
|  | **Precipitation_O,t_** | 1 |  |  | 8.223 | 0.008 |  |  |
|  | **Irrigation_Y,t_** | 1.49 |  |  | 0.699 | 0.485 |  |  |
| **22** | **Model** | 5.58 |  |  |  |  | 43.80% | 1.090 |
|  | **Intercept** | 1.00 | -3.83640 | -22.590 |  | < 0.001 |  |  |
|  | ***X*_T,t-1_** | 1.00 |  |  | 5.333 | 0.029 |  |  |
|  | **Ttotal** | 1.15 |  |  | 1.859 | 0.185 |  |  |
|  | **Precipitation_O,t_** | 1.00 |  |  | 8.170 | 0.008 |  |  |
|  | **Irrigation_Y,t_** | 1.58 |  |  | 0.717 | 0.492 |  |  |
| **23** | **Model** | 5.88 |  |  |  |  | 43.20% | 1.127 |
|  | **Intercept** | 1.00 | -3.83640 | -22.350 |  | < 0.001 |  |  |
|  | ***X*_T,t-1_** | 1.00 |  |  | 3.936 | 0.058 |  |  |
|  | **Tmean** | 1.11 |  |  | 0.655 | 0.442 |  |  |
|  | **Precipitation_O,t_** | 1.00 |  |  | 7.167 | 0.013 |  |  |
|  | **Irrigation_Y,t_** | 1.77 |  |  | 0.452 | 0.661 |  |  |
| **24** | **Model** | 5.49 |  |  |  |  | 41.50% | 1.127 |
|  | **Intercept** | 1.00 | -3.83600 | -22.180 |  | < 0.001 |  |  |
|  | ***X*_T,t-1_** | 1.00 |  |  | 4.716 | 0.039 |  |  |
|  | **Tmax** | 1.00 |  |  | 1.032 | 0.319 |  |  |
|  | **Precipitation_O,t_** | 1.00 |  |  | 5.941 | 0.022 |  |  |
|  | **Irrigation_Y,t_** | 1.49 |  |  | 0.330 | 0.702 |  |  |
| **25** | **Model** | 6.12 |  |  |  |  | 45.40% | 1.105 |
|  | **Intercept** | 1.00 | -3.83640 | -22.680 |  | < 0.001 |  |  |
|  | ***X*_T,t-1_** | 1.00 |  |  | 4.102 | 0.054 |  |  |
|  | **Tmin** | 1.29 |  |  | 1.207 | 0.290 |  |  |
|  | **Precipitation_O,t_** | 1.00 |  |  | 7.987 | 0.009 |  |  |
|  | **Irrigation_Y,t_** | 1.83 |  |  | 0.578 | 0.591 |  |  |
|  |  |  |  |  |  |  |  |  |
|  | **the first generation** | | | | | | | |
| **No.** | **Item** | DF | Estimate | t value | F value | p value | Dev.expl | GCV |
| **1** | **Model** | 2 |  |  |  |  | 1.01% | 1.633 |
|  | **Intercept** | 1 | 3.30900 | 14.910 |  | < 0.001 |  |  |
|  | ***X*_O,t_** | 1 |  |  | 0.295 | 0.591 |  |  |
| **2** | **Model** | 2 |  |  |  |  | 0.87% | 1.636 |
|  | **Intercept** | 1 | 3.30950 | 14.900 |  | < 0.001 |  |  |
|  | **Temperature_F,t_** | 1 |  |  | 0.255 | 0.617 |  |  |
| **3** | **Model** | 2 |  |  |  |  | 1.61% | 1.623 |
|  | **Intercept** | 1 | 3.30950 | 14.950 |  | < 0.001 |  |  |
|  | **Precipitation_F,t_** | 1 |  |  | 0.475 | 0.496 |  |  |
| **4** | **Model** | 2.87 |  |  |  |  | 25.70% | 1.303 |
|  | **Intercept** | 1 | 3.30950 | 16.950 |  | < 0.001 |  |  |
|  | **Irrigation_Y,t_** | 1.87 |  |  | 3.879 | 0.029 |  |  |
| **5** | **Model** | 3 |  |  |  |  | 2.21% | 1.731 |
|  | **Intercept** | 1 | 3.30950 | 14.740 |  | < 0.001 |  |  |
|  | ***X*_O,t_** | 1 |  |  | 0.383 | 0.541 |  |  |
|  | **Temperature_F,t_** | 1 |  |  | 0.344 | 0.562 |  |  |
| **6** | **Model** | 3 |  |  |  |  | 2.14% | 1.732 |
|  | **Intercept** | 1 | 3.30950 | 14.730 |  | < 0.001 |  |  |
|  | ***X*_O,t_** | 1 |  |  | 0.152 | 0.700 |  |  |
|  | **Precipitation_F,t_** | 1 |  |  | 0.324 | 0.574 |  |  |
| **7** | **Model** | 4.44 |  |  |  |  | 30.70% | 1.363 |
|  | **Intercept** | 1 | 3.30950 | 17.050 |  | < 0.001 |  |  |
|  | ***X*_O,t_** | 1.55 |  |  | 0.648 | 0.524 |  |  |
|  | **Irrigation_Y,t_** | 1.9 |  |  | 4.146 | 0.024 |  |  |
| **8** | **Model** | 3 |  |  |  |  | 4.25% | 1.695 |
|  | **Intercept** | 1 | 3.30950 | 14.890 |  | < 0.001 |  |  |
|  | **Temperature_F,t_** | 1 |  |  | 0.772 | 0.387 |  |  |
|  | **Precipitation_F,t_** | 1 |  |  | 0.988 | 0.329 |  |  |
| **9** | **Model** | 3.92 |  |  |  |  | 33.30% | 1.262 |
|  | **Intercept** | 1 | 3.30950 | 17.550 |  | < 0.001 |  |  |
|  | **Temperature_F,t_** | 1 |  |  | 2.846 | 0.103 |  |  |
|  | **Irrigation_Y,t_** | 1.92 |  |  | 5.233 | 0.010 |  |  |
| **10** | **Model** | 3.83 |  |  |  |  | 25.50% | 1.400 |
|  | **Intercept** | 1 | 3.30950 | 16.640 |  | < 0.001 |  |  |
|  | **Precipitation_F,t_** | 1 |  |  | 0.043 | 0.837 |  |  |
|  | **Irrigation_Y,t_** | 1.83 |  |  | 3.516 | 0.040 |  |  |
| **11** | **Model** | 4 |  |  |  |  | 4.93% | 1.810 |
|  | **Intercept** | 1 | 3.30950 | 14.680 |  | < 0.001 |  |  |
|  | ***X*_O,t_** | 1 |  |  | 0.192 | 0.664 |  |  |
|  | **Temperature_F,t_** | 1 |  |  | 0.792 | 0.381 |  |  |
|  | **Precipitation_F,t_** | 1 |  |  | 0.772 | 0.387 |  |  |
| **12** | **Model** | 4.94 |  |  |  |  | 34.90% | 1.331 |
|  | **Intercept** | 1 | 3.30900 | 17.420 |  | < 0.001 |  |  |
|  | ***X*_O,t_** | 1 |  |  | 0.451 | 0.508 |  |  |
|  | **Temperature_F,t_** | 1 |  |  | 2.846 | 0.103 |  |  |
|  | **Irrigation_Y,t_** | 1.94 |  |  | 5.081 | 0.011 |  |  |
| **13** | **Model** | 5.34 |  |  |  |  | 30.30% | 1.469 |
|  | **Intercept** | 1 | 3.30950 | 16.710 |  | < 0.001 |  |  |
|  | ***X*_O,t_** | 1.48 |  |  | 0.660 | 0.511 |  |  |
|  | **Precipitation_F,t_** | 1 |  |  | 0.103 | 0.751 |  |  |
|  | **Irrigation_Y,t_** | 1.86 |  |  | 3.859 | 0.031 |  |  |
| **14** | **Model** | 4.83 |  |  |  |  | 34.80% | 1.321 |
|  | **Intercept** | 1 | 3.30950 | 17.450 |  | < 0.001 |  |  |
|  | **Temperature_F,t_** | 1 |  |  | 3.824 | 0.061 |  |  |
|  | **Precipitation_F,t_** | 1 |  |  | 1.085 | 0.307 |  |  |
|  | **Irrigation_Y,t_** | 1.83 |  |  | 5.119 | 0.012 |  |  |
| **15** | **Model** | 5.86 |  |  |  |  | 37.40% | 1.374 |
|  | **Intercept** | 1 | 3.30950 | 17.450 |  | < 0.001 |  |  |
|  | ***X*_O,t_** | 1 |  |  | 0.871 | 0.360 |  |  |
|  | **Temperature_F,t_** | 1 |  |  | 4.204 | 0.051 |  |  |
|  | **Precipitation_F,t_** | 1 |  |  | 1.489 | 0.234 |  |  |
|  | **Irrigation_Y,t_** | 1.86 |  |  | 5.355 | 0.010 |  |  |
|  |  |  |  |  |  |  |  |  |
|  | **the second generation** | | | | | | | |
| **No.** | **Item** | DF | Estimate | t value | F value | p value | Dev.expl | GCV |
| **1** | **Model** | 3.71 |  |  |  |  | 41.70% | 1.155 |
|  | **Intercept** | 1 | -0.00267 | -0.015 |  | 0.988 |  |  |
|  | ***X*_F,t_** | 1 |  |  | 6.635 | 0.002 |  |  |
| **2** | **Model** | 2 |  |  |  |  | 0.20% | 1.751 |
|  | **Intercept** | 1 | -0.00267 | -0.012 |  | 0.991 |  |  |
|  | **Temperature_S,t_** | 1 |  |  | 0.059 | 0.809 |  |  |
| **3** | **Model** | 2 |  |  |  |  | 3.16% | 1.699 |
|  | **Intercept** | 1 | -0.00267 | -0.012 |  | 0.991 |  |  |
|  | **Precipitation_S,t_** | 1 |  |  | 0.948 | 0.338 |  |  |
| **4** | **Model** | 2 |  |  |  |  | 1.25% | 1.732 |
|  | **Intercept** | 1 | -0.00267 | -0.012 |  | 0.991 |  |  |
|  | **Irrigation_Y,t_** | 1 |  |  | 0.368 | 0.549 |  |  |
| **5** | **Model** | 4.69 |  |  |  |  | 41.60% | 1.244 |
|  | **Intercept** | 1 | -0.00267 | -0.014 |  | 0.989 |  |  |
|  | ***X*_F,t_** | 2.69 |  |  | 6.393 | 0.002 |  |  |
|  | **Temperature_S,t_** | 1 |  |  | 0.013 | 0.911 |  |  |
| **6** | **Model** | 4.76 |  |  |  |  | 44.10% | 1.197 |
|  | **Intercept** | 1 | -0.00267 | -0.015 |  | 0.988 |  |  |
|  | ***X*_F,t_** | 2.76 |  |  | 6.541 | 0.002 |  |  |
|  | **Precipitation_S,t_** | 1 |  |  | 0.884 | 0.356 |  |  |
| **7** | **Model** | 4.03 |  |  |  |  | 54.10% | 0.930 |
|  | **Intercept** | 1 | -0.00267 | -0.017 |  | 0.987 |  |  |
|  | ***X*_F,t_** | 1 |  |  | 28.339 | < 0.001 |  |  |
|  | **Irrigation_Y,t_** | 2.03 |  |  | 7.084 | 0.002 |  |  |
| **8** | **Model** | 3 |  |  |  |  | 3.83% | 1.810 |
|  | **Intercept** | 1 | -0.00267 | -0.012 |  | 0.991 |  |  |
|  | **Temperature_S,t_** | 1 |  |  | 0.193 | 0.664 |  |  |
|  | **Precipitation_S,t_** | 1 |  |  | 1.055 | 0.313 |  |  |
| **9** | **Model** | 3 |  |  |  |  | 1.31% | 1.857 |
|  | **Intercept** | 1 | -0.00267 | -0.011 |  | 0.991 |  |  |
|  | **Temperature_S,t_** | 1 |  |  | 0.015 | 0.902 |  |  |
|  | **Irrigation_Y,t_** | 1 |  |  | 0.313 | 0.580 |  |  |
| **10** | **Model** | 3 |  |  |  |  | 5.38% | 1.781 |
|  | **Intercept** | 1 | -0.00267 | -0.012 |  | 0.991 |  |  |
|  | **Precipitation_S,t_** | 1 |  |  | 1.222 | 0.278 |  |  |
|  | **Irrigation_Y,t_** | 1 |  |  | 0.657 | 0.425 |  |  |
| **11** | **Model** | 5.74 |  |  |  |  | 44.20% | 1.291 |
|  | **Intercept** | 1 | -0.00267 | -0.014 |  | 0.989 |  |  |
|  | ***X*_F,t_** | 2.74 |  |  | 6.218 | 0.003 |  |  |
|  | **Temperature_S,t_** | 1 |  |  | 0.078 | 0.783 |  |  |
|  | **Precipitation_S,t_** | 1 |  |  | 0.910 | 0.349 |  |  |
| **12** | **Model** | 6.81 |  |  |  |  | 63.70% | 0.916 |
|  | **Intercept** | 1 | -0.00267 | -0.018 |  | 0.986 |  |  |
|  | ***X*_F,t_** | 1 |  |  | 35.087 | < 0.001 |  |  |
|  | **Temperature_S,t_** | 2.69 |  |  | 1.856 | 0.164 |  |  |
|  | **Irrigation_Y,t_** | 2.12 |  |  | 9.273 | 0.001 |  |  |
| **13** | **Model** | 5.02 |  |  |  |  | 55.20% | 0.978 |
|  | **Intercept** | 1 | -0.00267 | -0.016 |  | 0.987 |  |  |
|  | ***X*_F,t_** | 1 |  |  | 26.128 | < 0.001 |  |  |
|  | **Precipitation_S,t_** | 1 |  |  | 0.691 | 0.413 |  |  |
|  | **Irrigation_Y,t_** | 2.02 |  |  | 7.130 | 0.002 |  |  |
| **14** | **Model** | 4 |  |  |  |  | 5.71% | 1.908 |
|  | **Intercept** | 1 | -0.00267 | -0.012 |  | 0.991 |  |  |
|  | **Temperature_S,t_** | 1 |  |  | 0.093 | 0.762 |  |  |
|  | **Precipitation_S,t_** | 1 |  |  | 1.261 | 0.271 |  |  |
|  | **Irrigation_Y,t_** | 1 |  |  | 0.539 | 0.469 |  |  |
| **15** | **Model** | 7.81 |  |  |  |  | 65.00% | 0.961 |
|  | **Intercept** | 1 | -0.00267 | -0.018 |  | 0.986 |  |  |
|  | ***X*_F,t_** | 1 |  |  | 31.876 | < 0.001 |  |  |
|  | **Temperature_S,t_** | 2.72 |  |  | 1.870 | 0.163 |  |  |
|  | **Precipitation_S,t_** | 1 |  |  | 0.858 | 0.364 |  |  |
|  | **Irrigation_Y,t_** | 2.09 |  |  | 9.176 | 0.001 |  |  |
|  |  |  |  |  |  |  |  |  |
|  | **the third generation** | | | | | | | |
| **No.** | **Item** | DF | Estimate | t value | F value | p value | Dev.expl | GCV |
| **1** | **Model** | 2 |  |  |  |  | 43.90% | 0.967 |
|  | **Intercept** | 1 | 0.58460 | 3.423 |  | 0.002 |  |  |
|  | ***X*_S,t_** | 1 |  |  | 22.660 | < 0.001 |  |  |
| **2** | **Model** | 2 |  |  |  |  | 11.90% | 1.517 |
|  | **Intercept** | 1 | 0.58460 | 2.733 |  | 0.011 |  |  |
|  | **Temperature_T,t_** | 1 |  |  | 3.927 | 0.057 |  |  |
| **3** | **Model** | 2 |  |  |  |  | 8.23% | 1.580 |
|  | **Intercept** | 1 | 0.58460 | 2.677 |  | 0.012 |  |  |
|  | **Precipitation_T,t_** | 1 |  |  | 2.599 | 0.118 |  |  |
| **4** | **Model** | 2 |  |  |  |  | 14.20% | 1.477 |
|  | **Intercept** | 1 | 0.58460 | 2.769 |  | 0.010 |  |  |
|  | **Irrigation_Y,t_** | 1 |  |  | 4.807 | 0.036 |  |  |
| **5** | **Model** | 3 |  |  |  |  | 57.70% | 0.782 |
|  | **Intercept** | 1 | 0.58460 | 3.874 |  | 0.001 |  |  |
|  | ***X*_S,t_** | 1 |  |  | 30.272 | < 0.001 |  |  |
|  | **Temperature_T,t_** | 1 |  |  | 9.139 | 0.005 |  |  |
| **6** | **Model** | 4.76 |  |  |  |  | 57.10% | 0.903 |
|  | **Intercept** | 1 | 0.58460 | 3.723 |  | 0.001 |  |  |
|  | ***X*_S,t_** | 1 |  |  | 27.279 | < 0.001 |  |  |
|  | **Precipitation_T,t_** | 2.76 |  |  | 1.985 | 0.141 |  |  |
| **7** | **Model** | 4.62 |  |  |  |  | 64.90% | 0.731 |
|  | **Intercept** | 1 | 0.58460 | 4.129 |  | < 0.001 |  |  |
|  | ***X*_S,t_** | 1 |  |  | 35.916 | < 0.001 |  |  |
|  | **Irrigation_Y,t_** | 2.62 |  |  | 4.617 | 0.011 |  |  |
| **8** | **Model** | 3 |  |  |  |  | 31.40% | 1.267 |
|  | **Intercept** | 1 | 0.58460 | 3.043 |  | 0.005 |  |  |
|  | **Temperature_T,t_** | 1 |  |  | 9.462 | 0.005 |  |  |
|  | **Precipitation_T,t_** | 1 |  |  | 7.951 | 0.009 |  |  |
| **9** | **Model** | 3 |  |  |  |  | 28.60% | 1.318 |
|  | **Intercept** | 1 | 0.58460 | 2.983 |  | 0.006 |  |  |
|  | **Temperature_T,t_** | 1 |  |  | 5.661 | 0.024 |  |  |
|  | **Irrigation_Y,t_** | 1 |  |  | 6.559 | 0.016 |  |  |
| **10** | **Model** | 3 |  |  |  |  | 18.30% | 1.510 |
|  | **Intercept** | 1 | 0.58460 | 2.787 |  | 0.009 |  |  |
|  | **Precipitation_T,t_** | 1 |  |  | 1.383 | 0.249 |  |  |
|  | **Irrigation_Y,t_** | 1 |  |  | 3.436 | 0.074 |  |  |
| **11** | **Model** | 4 |  |  |  |  | 65.00% | 0.696 |
|  | **Intercept** | 1 | 0.58460 | 4.180 |  | < 0.001 |  |  |
|  | ***X*_S,t_** | 1 |  |  | 25.846 | < 0.001 |  |  |
|  | **Temperature_T,t_** | 1 |  |  | 15.210 | 0.001 |  |  |
|  | **Precipitation_T,t_** | 1 |  |  | 5.603 | 0.025 |  |  |
| **12** | **Model** | 5.48 |  |  |  |  | 67.60% | 0.720 |
|  | **Intercept** | 1 | 0.58460 | 4.229 |  | < 0.001 |  |  |
|  | ***X*_S,t_** | 1 |  |  | 27.254 | < 0.001 |  |  |
|  | **Temperature_T,t_** | 1 |  |  | 3.121 | 0.089 |  |  |
|  | **Irrigation_Y,t_** | 2.48 |  |  | 2.132 | 0.123 |  |  |
| **13** | **Model** | 7.04 |  |  |  |  | 73.90% | 0.658 |
|  | **Intercept** | 1 | 0.58460 | 4.564 |  | < 0.001 |  |  |
|  | ***X*_S,t_** | 1 |  |  | 45.488 | < 0.001 |  |  |
|  | **Precipitation_T,t_** | 2.49 |  |  | 1.832 | 0.169 |  |  |
|  | **Irrigation_Y,t_** | 2.54 |  |  | 5.222 | 0.007 |  |  |
| **14** | **Model** | 4 |  |  |  |  | 41.40% | 1.164 |
|  | **Intercept** | 1 | 0.58460 | 3.233 |  | 0.003 |  |  |
|  | **Temperature_T,t_** | 1 |  |  | 10.674 | 0.003 |  |  |
|  | **Precipitation_T,t_** | 1 |  |  | 5.887 | 0.022 |  |  |
|  | **Irrigation_Y,t_** | 1 |  |  | 4.614 | 0.041 |  |  |
| **15** | **Model** | 6.41 |  |  |  |  | 75.00% | 0.598 |
|  | **Intercept** | 1 | 0.58460 | 4.725 |  | < 0.001 |  |  |
|  | ***X*_S,t_** | 1.01 |  |  | 29.733 | < 0.001 |  |  |
|  | **Temperature_T,t_** | 1 |  |  | 7.716 | 0.010 |  |  |
|  | **Precipitation_T,t_** | 1 |  |  | 7.665 | 0.010 |  |  |
|  | **Irrigation_Y,t_** | 2.4 |  |  | 2.873 | 0.060 |  |  |
