# Supplementary material for: Weakening density dependence from climate change and agricultural intensification triggers pest outbreaks: a 37-year observation of cotton bollworms
Source: Ecol Evol. 2014 Aug 12;4(17):3362–74. doi: 10.1002/ece3.1190 (PMC4228611; doi:10.1002/ece3.1190)
Supplement: Supplementary file 5 — Table S4. Main-effect linear candidate models (LM). [file ece30004-3362-sd5.doc]

**Table S4 Main-effect linear candidate models.** Main-effect population dynamic models (LM) fitted for adult cotton bollworm population of the whole year generation, the overwinter generation, the first generation, the second generation, the third generation.

|  | **the whole year generation** | | | | | | | |
| --- | --- | --- | --- | --- | --- | --- | --- | --- |
| **No.** | **Item** | DF | Estimate | t value | F value | p value | Dev.expl | GCV |
| **1** | **Model** | 2 |  |  | 5.385 | 0.028 | 15.70% | 1.049 |
|  | **Intercept** | 1 | 2.38600 | 2.334 |  | 0.027 |  |  |
|  | ***X*_Y,t-1_** | 1 | -0.27900 | -2.321 |  | 0.028 |  |  |
| **2** | **Model** | 2 |  |  | 0.131 | 0.720 | 0.45% | 1.238 |
|  | **Intercept** | 1 | 1.88120 | 0.372 |  | 0.713 |  |  |
|  | **Temperature_Y,t_** | 1 | -0.14400 | -0.362 |  | 0.720 |  |  |
| **3** | **Model** | 2 |  |  | 0.500 | 0.485 | 1.70% | 1.223 |
|  | **Intercept** | 1 | -0.41169 | -0.606 |  | 0.550 |  |  |
|  | **Precipitation_Y,t_** | 1 | 0.00092 | 0.707 |  | 0.485 |  |  |
| **4** | **Model** | 2 |  |  | 0.543 | 0.467 | 1.84% | 1.221 |
|  | **Intercept** | 1 | 0.70150 | 0.775 |  | 0.445 |  |  |
|  | **Irrigation_Y,t_** | 1 | -0.00003 | -0.737 |  | 0.467 |  |  |
| **5** | **Model** | 3 |  |  | 3.646 | 0.039 | 20.70% | 1.059 |
|  | **Intercept** | 1 | -4.32690 | -0.840 |  | 0.408 |  |  |
|  | ***X*_Y,t-1_** | 1 | -0.40200 | -2.671 |  | 0.013 |  |  |
|  | **Temperature_Y,t_** | 1 | 0.60880 | 1.329 |  | 0.195 |  |  |
| **6** | **Model** | 3 |  |  | 2.604 | 0.092 | 15.70% | 1.125 |
|  | **Intercept** | 1 | 2.30746 | 1.631 |  | 0.114 |  |  |
|  | ***X*_Y,t-1_** | 1 | -0.27588 | -2.155 |  | 0.040 |  |  |
|  | **Precipitation_Y,t_** | 1 | 0.00010 | 0.082 |  | 0.935 |  |  |
| **7** | **Model** | 3 |  |  | 3.084 | 0.062 | 18.10% | 1.093 |
|  | **Intercept** | 1 | 2.18000 | 2.075 |  | 0.047 |  |  |
|  | ***X*_Y,t-1_** | 1 | -0.37030 | -2.354 |  | 0.026 |  |  |
|  | **Irrigation_Y,t_** | 1 | < 0.00001 | 0.904 |  | 0.374 |  |  |
| **8** | **Model** | 3 |  |  | 0.243 | 0.786 | 1.71% | 1.312 |
|  | **Intercept** | 1 | -0.05751 | -0.009 |  | 0.992 |  |  |
|  | **Temperature_Y,t_** | 1 | -0.02634 | -0.059 |  | 0.953 |  |  |
|  | **Precipitation_Y,t_** | 1 | 0.00088 | 0.599 |  | 0.554 |  |  |
| **9** | **Model** | 3 |  |  | 0.264 | 0.770 | 1.85% | 1.310 |
|  | **Intercept** | 1 | 1.03300 | 0.195 |  | 0.847 |  |  |
|  | **Temperature_Y,t_** | 1 | -0.02810 | -0.064 |  | 0.950 |  |  |
|  | **Irrigation_Y,t_** | 1 | -0.00002 | -0.632 |  | 0.533 |  |  |
| **10** | **Model** | 3 |  |  | 0.410 | 0.668 | 2.84% | 1.296 |
|  | **Intercept** | 1 | 0.21430 | 0.166 |  | 0.869 |  |  |
|  | **Precipitation_Y,t_** | 1 | 0.00073 | 0.539 |  | 0.594 |  |  |
|  | **Irrigation_Y,t_** | 1 | -0.00002 | -0.575 |  | 0.570 |  |  |
| **11** | **Model** | 4 |  |  | 2.479 | 0.083 | 21.60% | 1.125 |
|  | **Intercept** | 1 | -5.95095 | -1.000 |  | 0.326 |  |  |
|  | ***X*_Y,t-1_** | 1 | -0.39899 | -2.617 |  | 0.014 |  |  |
|  | **Temperature_Y,t_** | 1 | 0.70452 | 1.427 |  | 0.165 |  |  |
|  | **Precipitation_Y,t_** | 1 | 0.00076 | 0.567 |  | 0.576 |  |  |
| **12** | **Model** | 4 |  |  | 2.663 | 0.068 | 22.80% | 1.107 |
|  | **Intercept** | 1 | -4.37400 | -0.845 |  | 0.405 |  |  |
|  | ***X*_Y,t-1_** | 1 | -0.48620 | -2.709 |  | 0.012 |  |  |
|  | **Temperature_Y,t_** | 1 | 0.59530 | 1.293 |  | 0.207 |  |  |
|  | **Irrigation_Y,t_** | 1 | < 0.00001 | 0.871 |  | 0.392 |  |  |
| **13** | **Model** | 4 |  |  | 1.991 | 0.139 | 18.10% | 1.175 |
|  | **Intercept** | 1 | 2.03700 | 1.403 |  | 0.172 |  |  |
|  | ***X*_Y,t-1_** | 1 | -0.36580 | -2.244 |  | 0.033 |  |  |
|  | **Precipitation_Y,t_** | 1 | 0.00019 | 0.146 |  | 0.885 |  |  |
|  | **Irrigation_Y,t_** | 1 | < 0.00001 | 0.896 |  | 0.378 |  |  |
| **14** | **Model** | 4 |  |  | 0.271 | 0.845 | 2.93% | 1.393 |
|  | **Intercept** | 1 | -0.71150 | -0.114 |  | 0.910 |  |  |
|  | **Temperature_Y,t_** | 1 | 0.07361 | 0.152 |  | 0.880 |  |  |
|  | **Precipitation_Y,t_** | 1 | 0.00081 | 0.547 |  | 0.589 |  |  |
|  | **Irrigation_Y,t_** | 1 | -0.00002 | -0.582 |  | 0.565 |  |  |
| **15** | **Model** | 5 |  |  | 2.048 | 0.117 | 24.00% | 1.177 |
|  | **Intercept** | 1 | -6.16800 | -1.032 |  | 0.312 |  |  |
|  | ***X*_Y,t-1_** | 1 | -0.48690 | -2.682 |  | 0.013 |  |  |
|  | **Temperature_Y,t_** | 1 | 0.70030 | 1.414 |  | 0.169 |  |  |
|  | **Precipitation_Y,t_** | 1 | 0.00083 | 0.622 |  | 0.540 |  |  |
|  | **Irrigation_Y,t_** | 1 | < 0.00001 | 0.899 |  | 0.377 |  |  |
|  |  |  |  |  |  |  |  |  |
|  | **the overwinter generation** | | | | | | | |
| **No.** | **Item** | DF | Estimate | t value | F value | p value | Dev.expl | GCV |
| **1** | **Model** | 2 |  |  | 6.906 | 0.014 | 19.20% | 1.204 |
|  | **Intercept** | 1 | -1.07040 | -1.001 |  | 0.325 |  |  |
|  | ***X*_T,t-1_** | 1 | -0.37600 | -2.628 |  | 0.014 |  |  |
| **2** | **Model** | 2 |  |  | 0.998 | 0.326 | 3.33% | 1.441 |
|  | **Intercept** | 1 | -4.50782 | -6.404 |  | 0.000 |  |  |
|  | **Ttotal** | 1 | -0.00289 | -0.999 |  | 0.326 |  |  |
| **3** | **Model** | 2 |  |  | 1.193 | 0.284 | 3.95% | 1.431 |
|  | **Intercept** | 1 | -2.35940 | -1.725 |  | 0.095 |  |  |
|  | **Tmean** | 1 | -0.29960 | -1.092 |  | 0.284 |  |  |
| **4** | **Model** | 2 |  |  | 0.015 | 0.902 | 0.05% | 1.489 |
|  | **Intercept** | 1 | -4.17562 | -1.523 |  | 0.139 |  |  |
|  | **Tmax** | 1 | 0.02952 | 0.124 |  | 0.902 |  |  |
| **5** | **Model** | 2 |  |  | 3.403 | 0.075 | 10.50% | 1.334 |
|  | **Intercept** | 1 | -3.88400 | -19.202 |  | < 0.001 |  |  |
|  | **Tmin** | 1 | -0.41270 | -1.845 |  | 0.075 |  |  |
| **6** | **Model** | 2 |  |  | 6.849 | 0.014 | 19.10% | 1.206 |
|  | **Intercept** | 1 | -2.73070 | -5.891 |  | 0.000 |  |  |
|  | **Precipitation_O,t_** | 1 | -0.01432 | -2.617 |  | 0.014 |  |  |
| **7** | **Model** | 2 |  |  | 1.281 | 0.267 | 4.23% | 1.427 |
|  | **Intercept** | 1 | -2.75400 | -2.813 |  | 0.009 |  |  |
|  | **Irrigation_Y,t_** | 1 | -< 0.00001 | -1.132 |  | 0.267 |  |  |
| **8** | **Model** | 3 |  |  | 3.536 | 0.043 | 20.20% | 1.276 |
|  | **Intercept** | 1 | -0.17992 | -0.095 |  | 0.925 |  |  |
|  | ***X*_T,t-1_** | 1 | -0.43698 | -2.430 |  | 0.022 |  |  |
|  | **Ttotal** | 1 | 0.00190 | 0.572 |  | 0.572 |  |  |
| **9** | **Model** | 3 |  |  | 3.459 | 0.045 | 19.80% | 1.282 |
|  | **Intercept** | 1 | -1.41900 | -1.064 |  | 0.296 |  |  |
|  | ***X*_T,t-1_** | 1 | -0.42400 | -2.353 |  | 0.026 |  |  |
|  | **Tmean** | 1 | 0.14240 | 0.450 |  | 0.657 |  |  |
| **10** | **Model** | 3 |  |  | 4.214 | 0.025 | 23.10% | 1.229 |
|  | **Intercept** | 1 | -3.70580 | -1.511 |  | 0.142 |  |  |
|  | ***X*_T,t-1_** | 1 | -0.44270 | -2.900 |  | 0.007 |  |  |
|  | **Tmax** | 1 | 0.27200 | 1.193 |  | 0.243 |  |  |
| **11** | **Model** | 3 |  |  | 3.389 | 0.048 | 19.50% | 1.287 |
|  | **Intercept** | 1 | -1.35395 | -0.937 |  | 0.357 |  |  |
|  | ***X*_T,t-1_** | 1 | -0.33877 | -1.768 |  | 0.088 |  |  |
|  | **Tmin** | 1 | -0.08483 | -0.298 |  | 0.768 |  |  |
| **12** | **Model** | 3 |  |  | 7.863 | 0.002 | 36.00% | 1.024 |
|  | **Intercept** | 1 | -0.20409 | -0.200 |  | 0.843 |  |  |
|  | ***X*_T,t-1_** | 1 | -0.35279 | -2.715 |  | 0.011 |  |  |
|  | **Precipitation_O,t_** | 1 | -0.01343 | -2.705 |  | 0.012 |  |  |
| **13** | **Model** | 3 |  |  | 3.444 | 0.046 | 19.70% | 1.283 |
|  | **Intercept** | 1 | -1.20300 | -1.065 |  | 0.296 |  |  |
|  | ***X*_T,t-1_** | 1 | -0.42170 | -2.326 |  | 0.028 |  |  |
|  | **Irrigation_Y,t_** | 1 | 0.00002 | 0.421 |  | 0.677 |  |  |
| **14** | **Model** | 3 |  |  | 3.526 | 0.043 | 20.10% | 1.277 |
|  | **Intercept** | 1 | -3.15835 | -3.688 |  | 0.001 |  |  |
|  | **Ttotal** | 1 | -0.00163 | -0.597 |  | 0.555 |  |  |
|  | **Precipitation_O,t_** | 1 | -0.01368 | -2.426 |  | 0.022 |  |  |
| **15** | **Model** | 3 |  |  | 0.918 | 0.411 | 6.16% | 1.500 |
|  | **Intercept** | 1 | -3.45100 | -2.558 |  | 0.016 |  |  |
|  | **Ttotal** | 1 | -0.00226 | -0.758 |  | 0.455 |  |  |
|  | **Irrigation_Y,t_** | 1 | -< 0.00001 | -0.919 |  | 0.366 |  |  |
| **16** | **Model** | 3 |  |  | 4.080 | 0.028 | 22.60% | 1.238 |
|  | **Intercept** | 1 | -1.77100 | -1.819 |  | 0.080 |  |  |
|  | **Precipitation_O,t_** | 1 | -0.01404 | -2.575 |  | 0.016 |  |  |
|  | **Irrigation_Y,t_** | 1 | -< 0.00001 | -1.119 |  | 0.273 |  |  |
| **17** | **Model** | 4 |  |  | 5.778 | 0.003 | 39.10% | 1.047 |
|  | **Intercept** | 1 | 1.52989 | 0.856 |  | 0.399 |  |  |
|  | ***X*_T,t-1_** | 1 | -0.46470 | -2.901 |  | 0.007 |  |  |
|  | **Ttotal** | 1 | 0.00355 | 1.179 |  | 0.249 |  |  |
|  | **Precipitation_O,t_** | 1 | -0.01455 | -2.897 |  | 0.007 |  |  |
| **18** | **Model** | 4 |  |  | 2.394 | 0.090 | 21.00% | 1.358 |
|  | **Intercept** | 1 | -0.18580 | -0.097 |  | 0.924 |  |  |
|  | ***X*_T,t-1_** | 1 | -0.50860 | -2.253 |  | 0.033 |  |  |
|  | **Ttotal** | 1 | 0.00226 | 0.658 |  | 0.516 |  |  |
|  | **Irrigation_Y,t_** | 1 | 0.00002 | 0.537 |  | 0.596 |  |  |
| **19** | **Model** | 4 |  |  | 5.177 | 0.006 | 36.50% | 1.091 |
|  | **Intercept** | 1 | -0.34080 | -0.318 |  | 0.753 |  |  |
|  | ***X*_T,t-1_** | 1 | -0.40040 | -2.436 |  | 0.022 |  |  |
|  | **Precipitation_O,t_** | 1 | -0.01345 | -2.671 |  | 0.013 |  |  |
|  | **Irrigation_Y,t_** | 1 | 0.00002 | 0.484 |  | 0.632 |  |  |
| **20** | **Model** | 4 |  |  | 2.678 | 0.067 | 22.90% | 1.325 |
|  | **Intercept** | 1 | -2.10600 | -1.545 |  | 0.134 |  |  |
|  | **Ttotal** | 1 | -0.00100 | -0.357 |  | 0.724 |  |  |
|  | **Precipitation_O,t_** | 1 | -0.01367 | -2.424 |  | 0.022 |  |  |
|  | **Irrigation_Y,t_** | 1 | -< 0.00001 | -0.992 |  | 0.330 |  |  |
| **21** | **Model** | 5 |  |  | 4.397 | 0.008 | 40.30% | 1.106 |
|  | **Intercept** | 1 | 1.54300 | 0.856 |  | 0.400 |  |  |
|  | ***X*_T,t-1_** | 1 | -0.55230 | -2.755 |  | 0.011 |  |  |
|  | **Ttotal** | 1 | 0.00400 | 1.292 |  | 0.208 |  |  |
|  | **Precipitation_O,t_** | 1 | -0.01472 | -2.903 |  | 0.007 |  |  |
|  | **Irrigation_Y,t_** | 1 | 0.00003 | 0.738 |  | 0.467 |  |  |
| **22** | **Model** | 5.00 |  |  | 4.397 | 0.008 | 40.30% | 1.106 |
|  | **Intercept** | 1.00 | 1.54300 | 0.856 |  | 0.400 |  |  |
|  | ***X*_T,t-1_** | 1.00 | -0.55230 | -2.755 |  | 0.011 |  |  |
|  | **Ttotal** | 1.00 | 0.00400 | 1.292 |  | 0.208 |  |  |
|  | **Precipitation_O,t_** | 1.00 | -0.01472 | -2.903 |  | 0.007 |  |  |
|  | **Irrigation_Y,t_** | 1.00 | 0.00003 | 0.738 |  | 0.467 |  |  |
| **23** | **Model** | 5.00 |  |  | 3.984 | 0.012 | 38.00% | 1.149 |
|  | **Intercept** | 1.00 | -0.88510 | -0.691 |  | 0.496 |  |  |
|  | ***X*_T,t-1_** | 1.00 | -0.48020 | -2.476 |  | 0.020 |  |  |
|  | **Tmean** | 1.00 | 0.22960 | 0.790 |  | 0.437 |  |  |
|  | **Precipitation_O,t_** | 1.00 | -0.01386 | -2.720 |  | 0.012 |  |  |
|  | **Irrigation_Y,t_** | 1.00 | 0.00002 | 0.511 |  | 0.613 |  |  |
| **24** | **Model** | 5.00 |  |  | 4.146 | 0.010 | 38.90% | 1.132 |
|  | **Intercept** | 1.00 | -2.60200 | -1.054 |  | 0.302 |  |  |
|  | ***X*_T,t-1_** | 1.00 | -0.47730 | -2.639 |  | 0.014 |  |  |
|  | **Tmax** | 1.00 | 0.22180 | 1.017 |  | 0.319 |  |  |
|  | **Precipitation_O,t_** | 1.00 | -0.01265 | -2.485 |  | 0.020 |  |  |
|  | **Irrigation_Y,t_** | 1.00 | 0.00003 | 0.684 |  | 0.500 |  |  |
| **25** | **Model** | 5.00 |  |  | 3.945 | 0.012 | 37.80% | 1.154 |
|  | **Intercept** | 1.00 | 0.48690 | 0.309 |  | 0.760 |  |  |
|  | ***X*_T,t-1_** | 1.00 | -0.47770 | -2.422 |  | 0.023 |  |  |
|  | **Tmin** | 1.00 | 0.20730 | 0.724 |  | 0.476 |  |  |
|  | **Precipitation_O,t_** | 1.00 | -0.01505 | -2.716 |  | 0.012 |  |  |
|  | **Irrigation_Y,t_** | 1.00 | 0.00001 | 0.366 |  | 0.717 |  |  |
|  |  |  |  |  |  |  |  |  |
|  | **the first generation** | | | | | | | |
| **No.** | **Item** | DF | Estimate | t value | F value | p value | Dev.expl | GCV |
| **1** | **Model** | 2 |  |  | 0.295 | 0.591 | 1.01% | 1.633 |
|  | **Intercept** | 1 | 2.98793 | 4.728 |  | 0.000 |  |  |
|  | ***X*_O,t_** | 1 | 0.09135 | 0.543 |  | 0.591 |  |  |
| **2** | **Model** | 2 |  |  | 0.255 | 0.617 | 0.87% | 1.636 |
|  | **Intercept** | 1 | 7.30530 | 0.923 |  | 0.364 |  |  |
|  | **Temperature_F,t_** | 1 | -0.17000 | -0.505 |  | 0.617 |  |  |
| **3** | **Model** | 2 |  |  | 0.475 | 0.496 | 1.61% | 1.623 |
|  | **Intercept** | 1 | 3.69378 | 6.155 |  | 0.000 |  |  |
|  | **Precipitation_F,t_** | 1 | -0.00271 | -0.689 |  | 0.496 |  |  |
| **4** | **Model** | 2 |  |  | 4.227 | 0.049 | 12.70% | 1.440 |
|  | **Intercept** | 1 | 1.33400 | 1.356 |  | 0.186 |  |  |
|  | **Irrigation_Y,t_** | 1 | 0.00008 | 2.056 |  | 0.049 |  |  |
| **5** | **Model** | 3 |  |  | 0.316 | 0.732 | 2.21% | 1.731 |
|  | **Intercept** | 1 | 7.67720 | 0.957 |  | 0.347 |  |  |
|  | ***X*_O,t_** | 1 | 0.10640 | 0.619 |  | 0.541 |  |  |
|  | **Temperature_F,t_** | 1 | -0.20170 | -0.586 |  | 0.562 |  |  |
| **6** | **Model** | 3 |  |  | 0.306 | 0.739 | 2.14% | 1.732 |
|  | **Intercept** | 1 | 3.40101 | 3.517 |  | 0.002 |  |  |
|  | ***X*_O,t_** | 1 | 0.06816 | 0.390 |  | 0.700 |  |  |
|  | **Precipitation_F,t_** | 1 | -0.00233 | -0.569 |  | 0.574 |  |  |
| **7** | **Model** | 3 |  |  | 2.101 | 0.141 | 13.00% | 1.539 |
|  | **Intercept** | 1 | 1.38700 | 1.370 |  | 0.182 |  |  |
|  | ***X*_O,t_** | 1 | -0.05750 | -0.324 |  | 0.748 |  |  |
|  | **Irrigation_Y,t_** | 1 | 0.00008 | 1.969 |  | 0.059 |  |  |
| **8** | **Model** | 3 |  |  | 0.622 | 0.544 | 4.25% | 1.695 |
|  | **Intercept** | 1 | 11.60356 | 1.286 |  | 0.209 |  |  |
|  | **Temperature_F,t_** | 1 | -0.32669 | -0.879 |  | 0.387 |  |  |
|  | **Precipitation_F,t_** | 1 | -0.00433 | -0.994 |  | 0.329 |  |  |
| **9** | **Model** | 3 |  |  | 3.187 | 0.057 | 18.50% | 1.442 |
|  | **Intercept** | 1 | 11.91000 | 1.580 |  | 0.125 |  |  |
|  | **Temperature_F,t_** | 1 | -0.47230 | -1.415 |  | 0.168 |  |  |
|  | **Irrigation_Y,t_** | 1 | 0.00010 | 2.465 |  | 0.020 |  |  |
| **10** | **Model** | 3 |  |  | 2.257 | 0.123 | 13.90% | 1.524 |
|  | **Intercept** | 1 | 1.69300 | 1.468 |  | 0.153 |  |  |
|  | **Precipitation_F,t_** | 1 | -0.00230 | -0.615 |  | 0.543 |  |  |
|  | **Irrigation_Y,t_** | 1 | 0.00008 | 1.998 |  | 0.056 |  |  |
| **11** | **Model** | 4 |  |  | 0.467 | 0.708 | 4.93% | 1.810 |
|  | **Intercept** | 1 | 11.50200 | 1.256 |  | 0.220 |  |  |
|  | ***X*_O,t_** | 1 | 0.07712 | 0.439 |  | 0.664 |  |  |
|  | **Temperature_F,t_** | 1 | -0.33618 | -0.890 |  | 0.381 |  |  |
|  | **Precipitation_F,t_** | 1 | -0.00395 | -0.879 |  | 0.387 |  |  |
| **12** | **Model** | 4 |  |  | 2.097 | 0.124 | 18.90% | 1.544 |
|  | **Intercept** | 1 | 11.99000 | 1.564 |  | 0.129 |  |  |
|  | ***X*_O,t_** | 1 | -0.05968 | -0.342 |  | 0.735 |  |  |
|  | **Temperature_F,t_** | 1 | -0.47340 | -1.395 |  | 0.174 |  |  |
|  | **Irrigation_Y,t_** | 1 | 0.00010 | 2.357 |  | 0.026 |  |  |
| **13** | **Model** | 4 |  |  | 1.539 | 0.227 | 14.60% | 1.626 |
|  | **Intercept** | 1 | 1.84100 | 1.522 |  | 0.140 |  |  |
|  | ***X*_O,t_** | 1 | -0.08748 | -0.476 |  | 0.638 |  |  |
|  | **Precipitation_F,t_** | 1 | -0.00274 | -0.701 |  | 0.489 |  |  |
|  | **Irrigation_Y,t_** | 1 | 0.00008 | 1.985 |  | 0.057 |  |  |
| **14** | **Model** | 4 |  |  | 2.864 | 0.055 | 24.10% | 1.444 |
|  | **Intercept** | 1 | 17.80000 | 2.094 |  | 0.046 |  |  |
|  | **Temperature_F,t_** | 1 | -0.69630 | -1.911 |  | 0.067 |  |  |
|  | **Precipitation_F,t_** | 1 | -0.00561 | -1.412 |  | 0.170 |  |  |
|  | **Irrigation_Y,t_** | 1 | 0.00010 | 2.661 |  | 0.013 |  |  |
| **15** | **Model** | 5 |  |  | 2.251 | 0.091 | 25.70% | 1.525 |
|  | **Intercept** | 1 | 18.82000 | 2.168 |  | 0.040 |  |  |
|  | ***X*_O,t_** | 1 | -0.13120 | -0.745 |  | 0.463 |  |  |
|  | **Temperature_F,t_** | 1 | -0.73100 | -1.973 |  | 0.059 |  |  |
|  | **Precipitation_F,t_** | 1 | -0.00642 | -1.546 |  | 0.134 |  |  |
|  | **Irrigation_Y,t_** | 1 | 0.00012 | 2.698 |  | 0.012 |  |  |
|  |  |  |  |  |  |  |  |  |
|  | **the second generation** | | | | | | | |
| **No.** | **Item** | DF | Estimate | t value | F value | p value | Dev.expl | GCV |
| **1** | **Model** | 2 |  |  | 8.091 | 0.008 | 21.80% | 1.372 |
|  | **Intercept** | 1 | 2.10790 | 2.740 |  | 0.010 |  |  |
|  | ***X*_F,t_** | 1 | -0.30900 | -2.845 |  | 0.008 |  |  |
| **2** | **Model** | 2 |  |  | 0.059 | 0.809 | 0.20% | 1.751 |
|  | **Intercept** | 1 | -1.62092 | -0.244 |  | 0.809 |  |  |
|  | **Temperature_S,t_** | 1 | 0.06121 | 0.244 |  | 0.809 |  |  |
| **3** | **Model** | 2 |  |  | 0.948 | 0.338 | 3.16% | 1.699 |
|  | **Intercept** | 1 | -0.42396 | -0.868 |  | 0.393 |  |  |
|  | **Precipitation_S,t_** | 1 | 0.00208 | 0.973 |  | 0.338 |  |  |
| **4** | **Model** | 2 |  |  | 0.368 | 0.549 | 1.25% | 1.732 |
|  | **Intercept** | 1 | -0.64200 | -0.595 |  | 0.556 |  |  |
|  | **Irrigation_Y,t_** | 1 | 0.00003 | 0.606 |  | 0.549 |  |  |
| **5** | **Model** | 3 |  |  | 3.984 | 0.030 | 22.20% | 1.465 |
|  | **Intercept** | 1 | 0.02367 | 0.004 |  | 0.997 |  |  |
|  | ***X*_F,t_** | 1 | -0.31011 | -2.810 |  | 0.009 |  |  |
|  | **Temperature_S,t_** | 1 | 0.07912 | 0.350 |  | 0.729 |  |  |
| **6** | **Model** | 3 |  |  | 4.056 | 0.028 | 22.50% | 1.459 |
|  | **Intercept** | 1 | 1.83350 | 1.902 |  | 0.068 |  |  |
|  | ***X*_F,t_** | 1 | -0.29744 | -2.640 |  | 0.013 |  |  |
|  | **Precipitation_S,t_** | 1 | 0.00096 | 0.484 |  | 0.632 |  |  |
| **7** | **Model** | 3 |  |  | 9.239 | 0.001 | 39.80% | 1.134 |
|  | **Intercept** | 1 | 0.45040 | 0.503 |  | 0.619 |  |  |
|  | ***X*_F,t_** | 1 | -0.48400 | -4.230 |  | < 0.001 |  |  |
|  | **Irrigation_Y,t_** | 1 | 0.00011 | 2.888 |  | 0.007 |  |  |
| **8** | **Model** | 3 |  |  | 0.557 | 0.579 | 3.83% | 1.810 |
|  | **Intercept** | 1 | -3.43799 | -0.500 |  | 0.621 |  |  |
|  | **Temperature_S,t_** | 1 | 0.11255 | 0.440 |  | 0.664 |  |  |
|  | **Precipitation_S,t_** | 1 | 0.00227 | 1.027 |  | 0.313 |  |  |
| **9** | **Model** | 3 |  |  | 0.185 | 0.832 | 1.31% | 1.857 |
|  | **Intercept** | 1 | -1.46400 | -0.217 |  | 0.829 |  |  |
|  | **Temperature_S,t_** | 1 | 0.03214 | 0.124 |  | 0.902 |  |  |
|  | **Irrigation_Y,t_** | 1 | 0.00002 | 0.559 |  | 0.580 |  |  |
| **10** | **Model** | 3 |  |  | 0.797 | 0.461 | 5.38% | 1.781 |
|  | **Intercept** | 1 | -1.36000 | -1.083 |  | 0.288 |  |  |
|  | **Precipitation_S,t_** | 1 | 0.00242 | 1.106 |  | 0.278 |  |  |
|  | **Irrigation_Y,t_** | 1 | 0.00003 | 0.810 |  | 0.425 |  |  |
| **11** | **Model** | 4 |  |  | 2.693 | 0.066 | 23.00% | 1.558 |
|  | **Intercept** | 1 | -0.96186 | -0.152 |  | 0.880 |  |  |
|  | ***X*_F,t_** | 1 | -0.29674 | -2.596 |  | 0.015 |  |  |
|  | **Temperature_S,t_** | 1 | 0.10419 | 0.447 |  | 0.659 |  |  |
|  | **Precipitation_S,t_** | 1 | 0.00114 | 0.555 |  | 0.584 |  |  |
| **12** | **Model** | 4 |  |  | 5.970 | 0.003 | 39.90% | 1.217 |
|  | **Intercept** | 1 | 1.70200 | 0.315 |  | 0.755 |  |  |
|  | ***X*_F,t_** | 1 | -0.48660 | -4.162 |  | < 0.001 |  |  |
|  | **Temperature_S,t_** | 1 | -0.04868 | -0.235 |  | 0.816 |  |  |
|  | **Irrigation_Y,t_** | 1 | 0.00011 | 2.821 |  | 0.009 |  |  |
| **13** | **Model** | 4 |  |  | 6.318 | 0.002 | 41.20% | 1.189 |
|  | **Intercept** | 1 | -0.01377 | -0.013 |  | 0.990 |  |  |
|  | ***X*_F,t_** | 1 | -0.47130 | -4.060 |  | < 0.001 |  |  |
|  | **Precipitation_S,t_** | 1 | 0.00147 | 0.828 |  | 0.415 |  |  |
|  | **Irrigation_Y,t_** | 1 | 0.00011 | 2.938 |  | 0.007 |  |  |
| **14** | **Model** | 4 |  |  | 0.545 | 0.656 | 5.71% | 1.908 |
|  | **Intercept** | 1 | -3.44300 | -0.497 |  | 0.623 |  |  |
|  | **Temperature_S,t_** | 1 | 0.08006 | 0.306 |  | 0.762 |  |  |
|  | **Precipitation_S,t_** | 1 | 0.00253 | 1.123 |  | 0.271 |  |  |
|  | **Irrigation_Y,t_** | 1 | 0.00003 | 0.734 |  | 0.469 |  |  |
| **15** | **Model** | 5 |  |  | 4.567 | 0.006 | 41.30% | 1.282 |
|  | **Intercept** | 1 | 0.48780 | 0.086 |  | 0.932 |  |  |
|  | ***X*_F,t_** | 1 | -0.47260 | -3.967 |  | 0.001 |  |  |
|  | **Temperature_S,t_** | 1 | -0.01914 | -0.090 |  | 0.929 |  |  |
|  | **Precipitation_S,t_** | 1 | 0.00144 | 0.783 |  | 0.440 |  |  |
|  | **Irrigation_Y,t_** | 1 | 0.00012 | 2.841 |  | 0.009 |  |  |
|  |  |  |  |  |  |  |  |  |
|  | **the third generation** | | | | | | | |
| **No.** | **Item** | DF | Estimate | t value | F value | p value | Dev.expl | GCV |
| **1** | **Model** | 2 |  |  | 22.660 | < 0.001 | 43.90% | 0.967 |
|  | **Intercept** | 1 | 3.85840 | 5.445 |  | < 0.001 |  |  |
|  | ***X*_S,t_** | 1 | -0.47950 | -4.761 |  | < 0.001 |  |  |
| **2** | **Model** | 2 |  |  | 3.927 | 0.057 | 11.90% | 1.517 |
|  | **Intercept** | 1 | -14.69460 | -1.905 |  | 0.067 |  |  |
|  | **Temperature_T,t_** | 1 | 0.64290 | 1.982 |  | 0.057 |  |  |
| **3** | **Model** | 2 |  |  | 2.599 | 0.118 | 8.23% | 1.580 |
|  | **Intercept** | 1 | -0.17394 | -0.335 |  | 0.740 |  |  |
|  | **Precipitation_T,t_** | 1 | 0.00266 | 1.612 |  | 0.118 |  |  |
| **4** | **Model** | 2 |  |  | 4.807 | 0.037 | 14.20% | 1.477 |
|  | **Intercept** | 1 | 2.71900 | 2.730 |  | 0.011 |  |  |
|  | **Irrigation_Y,t_** | 1 | -0.00008 | -2.192 |  | 0.037 |  |  |
| **5** | **Model** | 3 |  |  | 19.080 | 0.000 | 57.70% | 0.782 |
|  | **Intercept** | 1 | -12.52480 | -2.296 |  | 0.029 |  |  |
|  | ***X*_S,t_** | 1 | -0.49010 | -5.502 |  | < 0.001 |  |  |
|  | **Temperature_T,t_** | 1 | 0.69237 | 3.023 |  | 0.005 |  |  |
| **6** | **Model** | 3 |  |  | 11.550 | < 0.001 | 45.20% | 1.012 |
|  | **Intercept** | 1 | 3.38356 | 3.701 |  | 0.001 |  |  |
|  | ***X*_S,t_** | 1 | -0.45653 | -4.348 |  | < 0.001 |  |  |
|  | **Precipitation_T,t_** | 1 | 0.00112 | 0.829 |  | 0.414 |  |  |
| **7** | **Model** | 3 |  |  | 11.280 | < 0.001 | 44.60% | 1.023 |
|  | **Intercept** | 1 | 3.58400 | 4.247 |  | < 0.001 |  |  |
|  | ***X*_S,t_** | 1 | -0.53560 | -3.920 |  | 0.001 |  |  |
|  | **Irrigation_Y,t_** | 1 | 0.00003 | 0.616 |  | 0.543 |  |  |
| **8** | **Model** | 3 |  |  | 6.410 | 0.005 | 31.40% | 1.267 |
|  | **Intercept** | 1 | -23.50853 | -3.093 |  | 0.004 |  |  |
|  | **Temperature_T,t_** | 1 | 0.96107 | 3.076 |  | 0.005 |  |  |
|  | **Precipitation_T,t_** | 1 | 0.00439 | 2.820 |  | 0.009 |  |  |
| **9** | **Model** | 3 |  |  | 5.620 | 0.009 | 28.60% | 1.318 |
|  | **Intercept** | 1 | -13.96000 | -1.974 |  | 0.058 |  |  |
|  | **Temperature_T,t_** | 1 | 0.70970 | 2.379 |  | 0.024 |  |  |
|  | **Irrigation_Y,t_** | 1 | -0.00009 | -2.561 |  | 0.016 |  |  |
| **10** | **Model** | 3 |  |  | 3.127 | 0.059 | 18.30% | 1.510 |
|  | **Intercept** | 1 | 1.88500 | 1.548 |  | 0.133 |  |  |
|  | **Precipitation_T,t_** | 1 | 0.00192 | 1.176 |  | 0.250 |  |  |
|  | **Irrigation_Y,t_** | 1 | -0.00007 | -1.854 |  | 0.074 |  |  |
| **11** | **Model** | 4 |  |  | 16.680 | < 0.001 | 65.00% | 0.696 |
|  | **Intercept** | 1 | -18.35688 | -3.264 |  | 0.003 |  |  |
|  | ***X*_S,t_** | 1 | -0.43566 | -5.084 |  | < 0.001 |  |  |
|  | **Temperature_T,t_** | 1 | 0.88871 | 3.900 |  | 0.001 |  |  |
|  | **Precipitation_T,t_** | 1 | 0.00279 | 2.367 |  | 0.025 |  |  |
| **12** | **Model** | 4 |  |  | 12.430 | < 0.001 | 58.00% | 0.834 |
|  | **Intercept** | 1 | -12.50000 | -2.258 |  | 0.032 |  |  |
|  | ***X*_S,t_** | 1 | -0.52650 | -4.344 |  | < 0.001 |  |  |
|  | **Temperature_T,t_** | 1 | 0.68370 | 2.933 |  | 0.007 |  |  |
|  | **Irrigation_Y,t_** | 1 | 0.00002 | 0.451 |  | 0.656 |  |  |
| **13** | **Model** | 4 |  |  | 7.719 | 0.001 | 46.20% | 1.069 |
|  | **Intercept** | 1 | 3.03400 | 2.884 |  | 0.008 |  |  |
|  | ***X*_S,t_** | 1 | -0.51840 | -3.742 |  | 0.001 |  |  |
|  | **Precipitation_T,t_** | 1 | 0.00120 | 0.882 |  | 0.385 |  |  |
|  | **Irrigation_Y,t_** | 1 | 0.00003 | 0.694 |  | 0.494 |  |  |
| **14** | **Model** | 4 |  |  | 6.363 | 0.002 | 41.40% | 1.164 |
|  | **Intercept** | 1 | -21.44000 | -2.971 |  | 0.006 |  |  |
|  | **Temperature_T,t_** | 1 | 0.96070 | 3.267 |  | 0.003 |  |  |
|  | **Precipitation_T,t_** | 1 | 0.00365 | 2.426 |  | 0.022 |  |  |
|  | **Irrigation_Y,t_** | 1 | -0.00007 | -2.148 |  | 0.041 |  |  |
| **15** | **Model** | 5 |  |  | 12.350 | < 0.001 | 65.50% | 0.739 |
|  | **Intercept** | 1 | -18.43000 | -3.242 |  | 0.003 |  |  |
|  | ***X*_S,t_** | 1 | -0.48330 | -4.263 |  | < 0.001 |  |  |
|  | **Temperature_T,t_** | 1 | 0.88090 | 3.819 |  | 0.001 |  |  |
|  | **Precipitation_T,t_** | 1 | 0.00284 | 2.381 |  | 0.025 |  |  |
|  | **Irrigation_Y,t_** | 1 | 0.00002 | 0.651 |  | 0.521 |  |  |
