# Supplementary material for: Weakening density dependence from climate change and agricultural intensification triggers pest outbreaks: a 37-year observation of cotton bollworms
Source: Ecol Evol. 2014 Aug 12;4(17):3362–74. doi: 10.1002/ece3.1190 (PMC4228611; doi:10.1002/ece3.1190)
Supplement: Supplementary file 6 — Table S5. Candidate models with interactions. [file ece30004-3362-sd6.doc]

**Table S5. Candidate models with interactions.** Models are fitted for adult cotton bollworm population of the overwinter generation, the first generation, the second generation, the third generation. Linear candidate models are list with lower GCV than respect season main-effect linear models. The annotations are the same as above. X, T, P and I indicate natural log-transformed population density, temperature, precipitation and irrigation area. “×” indicates interaction.

|  | **the overwinter generation** | | | | | | | |
| --- | --- | --- | --- | --- | --- | --- | --- | --- |
| **No.** | **Item** | DF | Estimate | t value | F value | p value | Dev.expl | GCV |
| **1** | **Model** | 3 |  |  | 8.289 | 0.001 | 37.20% | 1.004 |
|  | **Intercept** | 1 | 0.89530 | 0.755 |  | 0.456 |  |  |
|  | **X** | 1 | -0.50030 | -3.687 |  | 0.001 |  |  |
|  | **T×P** | 1 | 0.00006 | 2.829 |  | 0.009 |  |  |
|  |  |  |  |  |  |  |  |  |
|  | **the first generation** | | | | | | | |
| **No.** | **Item** | DF | Estimate | t value | F value | p value | Dev.expl | GCV |
| **1** | **Model** | 5 |  |  | 2.933 | 0.040 | 31.10% | 1.415 |
|  | **Intercept** | 1 | 0.74880 | 0.774 |  | 0.446 |  |  |
|  | **X** | 1 | 6.50300 | 2.620 |  | 0.014 |  |  |
|  | **X×T** | 1 | -0.28390 | -2.649 |  | 0.014 |  |  |
|  | **T×I** | 1 | 0.00001 | 3.238 |  | 0.003 |  |  |
|  | **P×I** | 1 | < 0.00001 | -1.828 |  | 0.079 |  |  |
| **2** | **Model** | 5 |  |  | 2.908 | 0.041 | 30.90% | 1.418 |
|  | **Intercept** | 1 | 0.60560 | 0.620 |  | 0.541 |  |  |
|  | **X** | 1 | 6.48700 | 2.609 |  | 0.015 |  |  |
|  | **X×T** | 1 | -0.27370 | -2.605 |  | 0.015 |  |  |
|  | **X×P** | 1 | -0.00196 | -1.807 |  | 0.082 |  |  |
|  | **T×I** | 1 | 0.00001 | 3.123 |  | 0.004 |  |  |
| **3** | **Model** | 5 |  |  | 2.806 | 0.046 | 30.20% | 1.434 |
|  | **Intercept** | 1 | 0.72730 | 0.737 |  | 0.468 |  |  |
|  | **X** | 1 | 5.35000 | 2.304 |  | 0.029 |  |  |
|  | **I** | 1 | 0.00016 | 3.161 |  | 0.004 |  |  |
|  | **X×T** | 1 | -0.23460 | -2.346 |  | 0.027 |  |  |
|  | **P×I** | 1 | < 0.00001 | -1.819 |  | 0.081 |  |  |
| **4** | **Model** | 5 |  |  | 2.787 | 0.047 | 30% | 1.437 |
|  | **Intercept** | 1 | 0.59750 | 0.599 |  | 0.554 |  |  |
|  | **X** | 1 | 5.50900 | 2.330 |  | 0.028 |  |  |
|  | **I** | 1 | 0.00014 | 3.049 |  | 0.005 |  |  |
|  | **X×T** | 1 | -0.23170 | -2.326 |  | 0.028 |  |  |
|  | **X×P** | 1 | -0.00197 | -1.802 |  | 0.083 |  |  |
| **5** | **Model** | 5 |  |  | 2.783 | 0.048 | 30% | 1.437 |
|  | **Intercept** | 1 | 18.75000 | 2.390 |  | 0.024 |  |  |
|  | **X** | 1 | 1.56400 | 1.868 |  | 0.073 |  |  |
|  | **T** | 1 | -1.02100 | -2.537 |  | 0.018 |  |  |
|  | **X×I** | 1 | -0.00006 | -1.978 |  | 0.059 |  |  |
|  | **T×I** | 1 | 0.00002 | 2.667 |  | 0.013 |  |  |
|  |  |  |  |  |  |  |  |  |
|  | **the second generation** | | | | | | | |
| **No.** | **Item** | DF | Estimate | t value | F value | p value | Dev.expl | GCV |
| **1** | **Model** | 5 |  |  | 6.092 | 0.001 | 48.40% | 1.127 |
|  | **Intercept** | 1 | -4.04400 | -3.525 |  | 0.002 |  |  |
|  | **P** | 1 | 0.05828 | 2.243 |  | 0.034 |  |  |
|  | **X×I** | 1 | -0.00002 | -4.557 |  | < 0.001 |  |  |
|  | **T×P** | 1 | -0.00210 | -2.179 |  | 0.039 |  |  |
|  | **T×I** | 1 | 0.00001 | 4.563 |  | < 0.001 |  |  |
| **2** | **Model** | 3 |  |  | 10.570 | < 0.001 | 43% | 1.072 |
|  | **Intercept** | 1 | -3.09400 | -3.112 |  | 0.004 |  |  |
|  | **I** | 1 | 0.00027 | 4.310 |  | < 0.001 |  |  |
|  | **X×I** | 1 | -0.00002 | -4.531 |  | < 0.001 |  |  |
|  |  |  |  |  |  |  |  |  |
|  | **the third generation** | | | | | | | |
| **No.** | **Item** | DF | Estimate | t value | F value | p value | Dev.expl | GCV |
| **1** | **Model** | 4 |  |  | 17.290 | < 0.001 | 65.80% | 0.680 |
|  | **Intercept** | 1 | -21.62000 | -3.930 |  | 0.001 |  |  |
|  | **T** | 1 | 1.06000 | 4.594 |  | < 0.001 |  |  |
|  | **X×T** | 1 | -0.02339 | -6.493 |  | < 0.001 |  |  |
|  | **X×P** | 1 | 0.00043 | 2.450 |  | 0.021 |  |  |
| **2** | **Model** | 4 |  |  | 17.150 | < 0.001 | 65.60% | 0.684 |
|  | **Intercept** | 1 | -20.68000 | -3.849 |  | 0.001 |  |  |
|  | **T** | 1 | 0.98690 | 4.456 |  | < 0.001 |  |  |
|  | **X×T** | 1 | -0.01845 | -5.149 |  | < 0.001 |  |  |
|  | **T×P** | 1 | 0.00012 | 2.414 |  | 0.023 |  |  |
| **3** | **Model** | 4 |  |  | 17.000 | < 0.001 | 65.40% | 0.688 |
|  | **Intercept** | 1 | -17.81000 | -3.258 |  | 0.003 |  |  |
|  | **X** | 1 | -0.55290 | -6.433 |  | < 0.001 |  |  |
|  | **T** | 1 | 0.89880 | 3.957 |  | < 0.001 |  |  |
|  | **X×P** | 1 | 0.00043 | 2.451 |  | 0.021 |  |  |
| **4** | **Model** | 4 |  |  | 16.840 | < 0.001 | 65.20% | 0.692 |
|  | **Intercept** | 1 | -17.68000 | -3.229 |  | 0.003 |  |  |
|  | **X** | 1 | -0.43460 | -5.087 |  | < 0.001 |  |  |
|  | **T** | 1 | 0.85920 | 3.860 |  | 0.001 |  |  |
|  | **T×P** | 1 | 0.00012 | 2.411 |  | 0.023 |  |  |
| **6** | **Model** | 4 |  |  | 16.990 | < 0.001 | 65.40% | 0.688 |
|  | **Intercept** | 1 | -21.36786 | -3.875 |  | 0.001 |  |  |
|  | **T** | 1 | 1.01670 | 4.493 |  | < 0.001 |  |  |
|  | **P** | 1 | 0.00278 | 2.373 |  | 0.025 |  |  |
|  | **X×T** | 1 | -0.01850 | -5.147 |  | < 0.001 |  |  |
